# Discontinuation of oral anticoagulants is common after catheter ablation in older patients with atrial fibrillation

**DOI:** 10.1093/ehjopen/oeag128

**Published:** 2026-08-18

**Authors:** Xiaojuan Liu, Daniel E Singer, E Kevin Heist, Kueiyu Joshua Lin

**Affiliations:** Division of Pharmacoepidemiology and Pharmacoeconomics, Department of Medicine, Brigham and Women’s Hospital, Harvard Medical School, 399 Revolution Drive, Somerville, Boston, MA 02145, USA; Division of General Internal Medicine, Department of Medicine, Massachusetts General Hospital, Harvard Medical School, 100 Cambridge Street, Boston, MA 02114, USA; Demoulas Center for Cardiac Arrhythmias, Massachusetts General Hospital, Harvard Medical School, 55 Fruit Street, Boston, MA 02114, USA; Division of Pharmacoepidemiology and Pharmacoeconomics, Department of Medicine, Brigham and Women’s Hospital, Harvard Medical School, 399 Revolution Drive, Somerville, Boston, MA 02145, USA

**Keywords:** Atrial fibrillation, Oral anticoagulation, Ablation

## Abstract

**Aims:**

Guidelines recommend continuing oral anticoagulation (OAC) after atrial fibrillation (AF) ablation based on stroke risk, yet real-world post-procedural OAC management remains poorly characterized. We aimed to evaluate contemporary patterns of OAC use after AF ablation and identify predictors of OAC discontinuation.

**Methods and results:**

Using Medicare claims from 2013–2022, we established a cohort of patients undergoing a first AF ablation and assessed patterns of OAC discontinuation during the subsequent year. We defined OAC discontinuation as a dispensing gap ≥60 days. Baseline characteristics were evaluated as predictors of discontinuation using least absolute shrinkage and selection operator Cox regression. Among 208 232 AF patients (mean age 74.5, 47% women) undergoing ablation, 58% were taking OAC following ablation, 24% discontinued between 3 and 12 months, 8% discontinued within 3 months, and 10% did not use OAC within 3 months. Apixaban was the most commonly used OAC agent, while the use of warfarin declined over time. The overall rate of repeated ablation was 26%. We found that 44% of patients with CHA_2_DS_2_-VASc score ≥3 discontinued OAC within 1 year after ablation. Higher CHA_2_DS_2_-VASc scores and longer pre-ablation OAC use were associated with lower discontinuation, whereas pre-ablation major bleeding, frailty, and use of warfarin (vs. direct OACs) increased the likelihood of discontinuation.

**Conclusion:**

OAC discontinuation after AF ablation is common, including among patients at elevated stroke risk despite guideline recommendations for continued therapy. More data are needed on the clinical consequences of post-ablation OAC discontinuation.

## Graphical Abstract

**Graphical Abstract:**
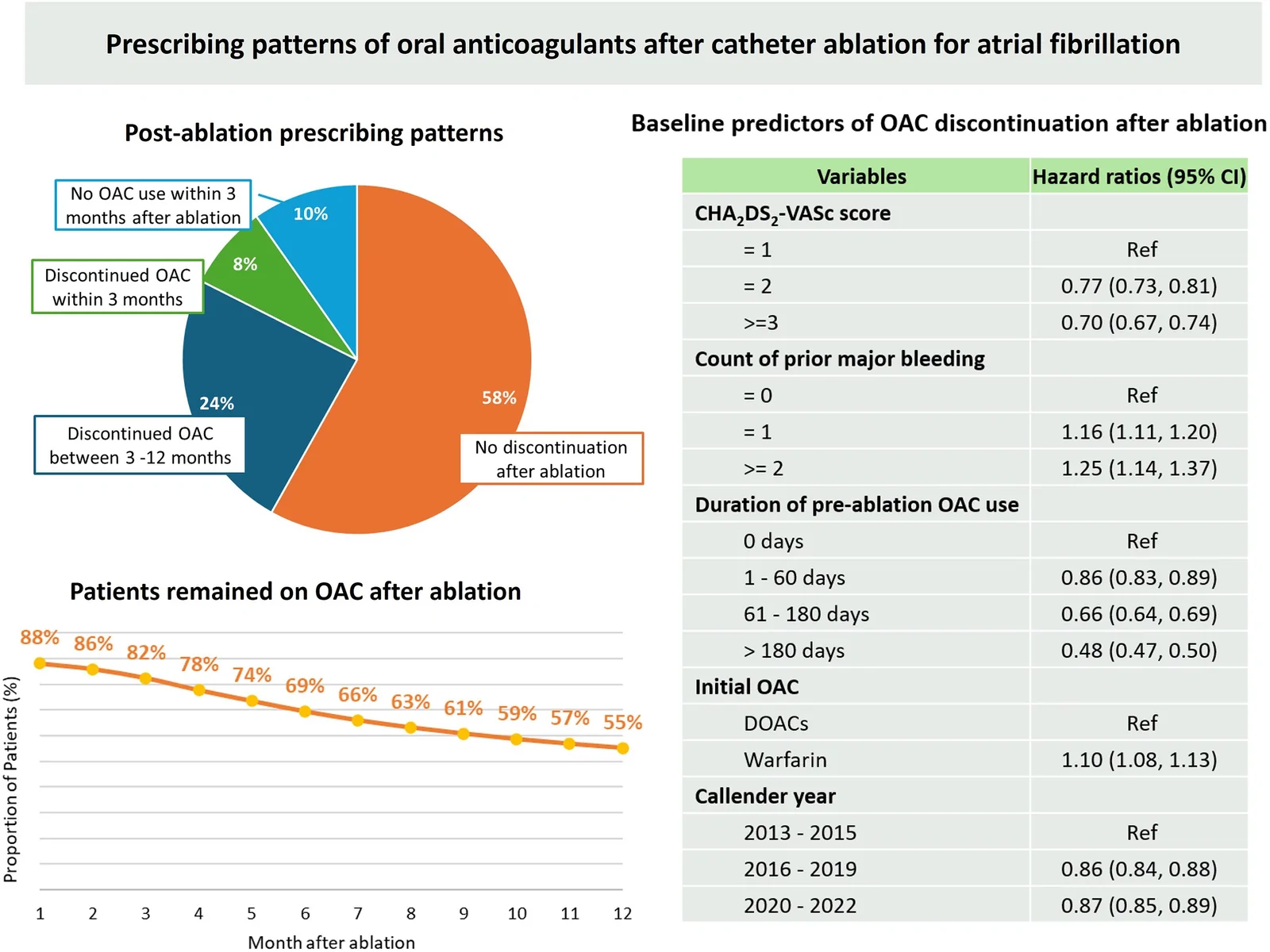

## Introduction

Atrial fibrillation (AF) is the most prevalent cardiac arrhythmia and a major risk factor for stroke.^1,2^ Catheter ablation is an established cardiac procedure used for rhythm control in patients with AF. Randomized clinical trials have shown that ablation reduces arrhythmia burden, improves quality of life, and enhances cardiovascular outcomes.^3–7^ However, ablation does not eliminate AF in all patients.^8^ The risk of stroke in the first 30 days after ablation is 0.8%,^9^ and AF recurrences, often asymptomatic, are frequent.^10^ Consequently, oral anticoagulation (OAC) after catheter ablation procedures is generally recommended.^2^

The optimal need and duration of anticoagulation after ablation remain uncertain, and substantial practice variation persists. For instance, a US cohort study of 6886 AF patients who underwent AF ablation between 2005 and 2014 reported OAC discontinuation rates of 39.5% at 3 months and 68.7% at 12 months post-procedure,^11^ while a US registry including 21 595 AF patients from 2011 to 2016 found that 23% discontinued OAC, with a median time to discontinuation of 6 months post-ablation.^12^ Current guidelines recommend maintaining OAC for at least three months after ablation, with continued therapy thereafter guided by individual stroke risk,^2^ yet real-world treatment patterns and adherence to these recommendations are unclear. A better understanding of the current practices of various post-ablation antithrombotic regimens is critical to inform optimal management approaches.

To address these gaps, we aimed to use a US nationwide health insurance claims data to characterize patterns of OAC use in patients who underwent catheter ablation for atrial fibrillation, including rates and timing of OAC discontinuation, and identify factors associated with anticoagulation discontinuation in routine clinical practice.

## Methods

### Study population

We used data from US Medicare fee-for-service Parts A (inpatient), B (outpatient), and D (pharmacy claims) from 1 January 2013 to 31 December 2022, to establish the study cohort. This public health insurance covers patients aged 65 years or older and those with disability. It provides comprehensive longitudinal, patient-level data, including inpatient and outpatient diagnoses, procedures, and all dispensed prescription medications. The study was approved by the Brigham and Women’s Hospital Institutional Review Board.

We included participants who received their first catheter ablation after being diagnosed with non-valvular AF. Patients were required to have continuous enrolment for at least 12 months prior to ablation. The date of the ablation was defined as the cohort entry (index) date. Patients were excluded if they (1) were aged <65 years; (2) required anticoagulation for reasons other than AF, such as venous thromboembolism (VTE; deep vein thrombosis or pulmonary embolism); (3) had ‘valvular heart disease’, i.e. mitral stenosis, rheumatic mitral insufficiency, mitral stenosis with insufficiency, or mechanical heart valve replacement; (4) had end-stage renal disease, end-stage liver disease, or malignant neoplasms (excluding non-melanoma skin cancer); or (5) were enroled in hospice or palliative care. *Figure 1* shows a flowchart of applying these eligibility criteria to the study population (event definitions in Supplementary Method).

**Figure 1:**
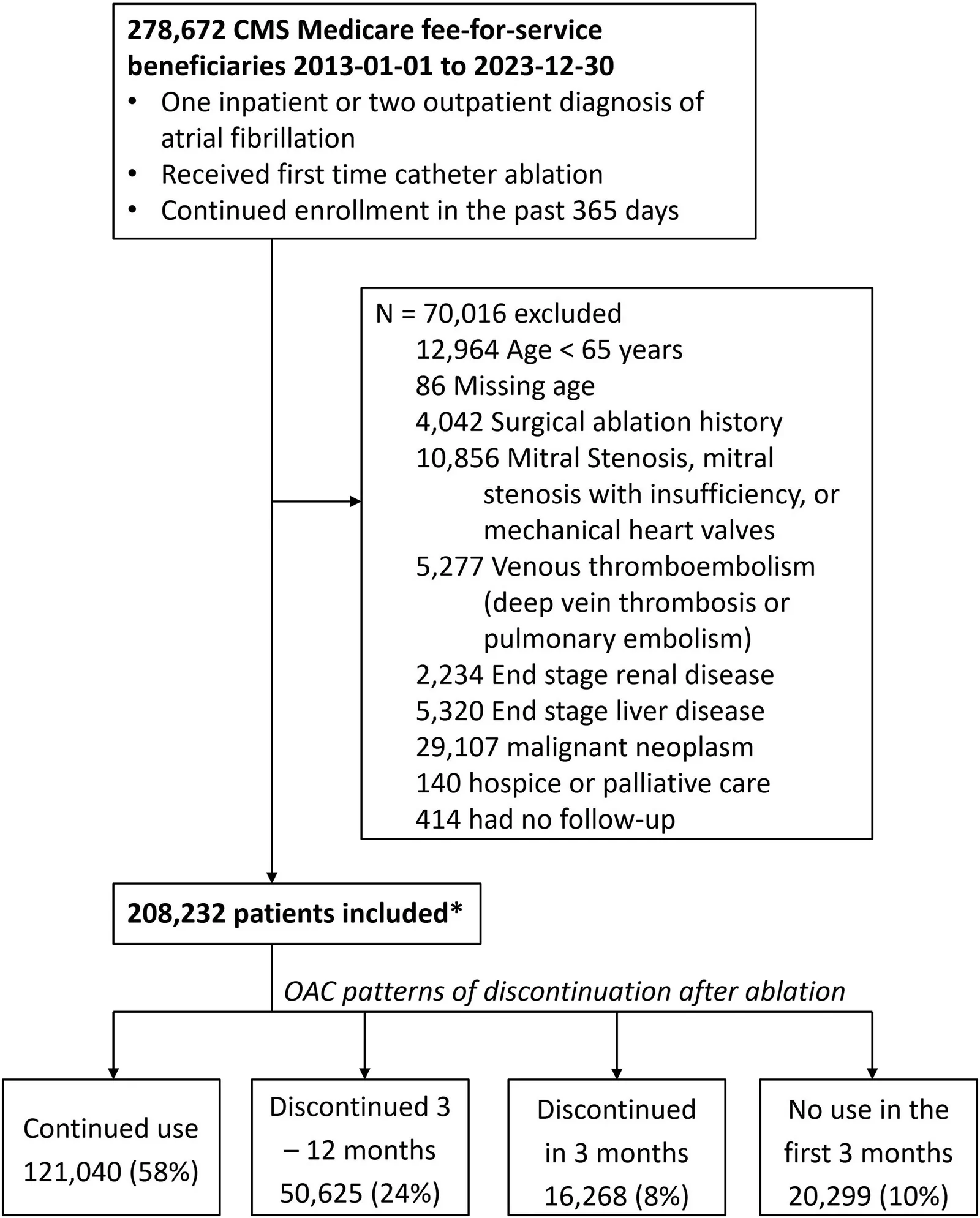
Flowchart of study population. *Among all included patients, 185 838 (89%) patients used oral anticoagulants in one year prior to the index ablation.

### Measurements

We used the 365 days before (and including) the index date as the baseline assessment period to measure covariates, including demographics, geographic region, comorbidities, medication use, calendar year, CHA_2_DS_2_-VASc score (congestive heart failure, hypertension, age ≥ 75 years, diabetes, stroke or transient ischaemic attack, vascular disease, age 65–74 years, and sex), count of major bleeding events and HAS-BLED score (hypertension, abnormal liver and/or kidney function, stroke history, bleeding history or predisposition, labile international normalized ratio, elderly, drug and alcohol use concomitantly).^13^ The burden of comorbidity was quantified via a combined comorbidity score.^14^ Frailty was measured using a validated claims-based frailty index (CFI) and categorized into non-frail (CFI < 0.15), pre-frail (CFI: 0.15 to <0.25), and frail (CFI ≥ 0.25).^15,16^ Covariates were identified using claims-based diagnosis codes, procedure codes, and National Drug Codes.

### Follow-up and outcomes

Patients were followed up for 12 months to capture post-procedural treatments. We categorized patients based on post-procedural anticoagulation patterns and divided them into four mutually exclusive subgroups: (1) no discontinuation, (2) discontinued between 3 and 12 months, (3) discontinued within 3 months, and (4) no use within 3 months. The cut-off at 3 months was chosen based on the current guideline recommendation for continued oral anticoagulation for at least 3 months after catheter ablation.^2^ Post-ablation OAC use was ascertained using pharmacy dispensing data and therefore reflects medication fills rather than discharge prescriptions. Medication discontinuation was defined by an absence of refill in 60 days (dispensing gap ≥ 60 days). We described the median length of OAC use post-ablation and rate of OAC reinitiation in the discontinuation groups. We then assessed use of post-ablation antiarrhythmic and rate control medications, cardioversion, repeated ablation, and clinical outcomes including hospitalization for major bleeding, hospitalization for ischaemic stroke, and all-cause mortality. Outcome definitions based on ICD codes are provided in Supplementary Method.

### Statistical analysis

Baseline characteristics were assessed in the overall sample and by distinct therapy pattern groups. We reported the trend of initial OAC choice over calendar time as well as the proportion of patients remained on anticoagulation at each month in the subsequent year. To identify factors associated with discontinuation of anticoagulation, we applied the least absolute shrinkage and selection operator (LASSO) Cox regression via the ‘glmnet’ R package. We excluded patients who did not use OAC within 3 months after ablation. The primary outcome was time to discontinuation between 3 and 12 months after ablation vs. continued use. We used ten-fold cross-validation based on the mean squared error (MSE) criterion to determine the optimal penalty regularization parameter (lambda). To prevent overfitting, we fixed lambda.1se, the largest lambda at which the MSE remained within one standard error of the minimum MSE. As this analysis was agnostic, all available baseline variables were included with equal penalization. Hazard ratios (HRs) and 95% confidence intervals (CIs) for LASSO-selected predictors were subsequently estimated using multivariable Cox regression, adjusting for all selected variables. In the secondary analyses, we (1) used a secondary outcome of time to OAC discontinuation at any time post-ablation vs. continued use and (2) evaluated the primary outcome in patients with elevated stroke risk (defined by a CHA_2_DS_2_-VASc score ≥2 for men or ≥3 for women). Additionally, sensitivity analyses were performed using alternative medication discontinuation definitions based on dispensing gaps of 30 and 90 days. All analyses were performed in the Aetion Evidence Generation Platform (https://www.aetion.com) and R software, version 4.4.1.

## Results

### Baseline characteristics

Our study population included 208 232 AF patients (mean [SD] age = 74.5 [6], 47% women; mean CHA_2_DS_2_-VASc score = 4.0 [1.5]) who underwent AF ablation between 2013 and 2022. A total of 121 040 (58%) patients were taking OAC with no discontinuation after ablation, 50 625 (24%) discontinued between 3 and 12 months, 16 268 (8%) discontinued within 3 months, and 20 299 (10%) did not use OAC within the first 3 months. Compared to patients who continued OAC for at least 3 months, patients who discontinued within 3 months or did not use OAC were older, had a higher CHA_2_DS_2_-VASc score, and more pre-ablation bleeding events (*Table 1*). By contrast, patients who discontinued between 3 and 12 months were younger, had lower CHA_2_DS_2_-VASc scores and lower rates of comorbidities. Notably, among patients with no OAC use within three months, most had not been receiving anticoagulation prior to ablation and demonstrated the highest use of P2Y_12_ inhibitors. A complete list of baseline characteristics is shown in Supplementary material online, *Table S1*.

**Table 1:** Pre-ablation characteristics of the study population by OAC pattern groups

Characteristics	Continued use^a^ *N* = 121 040	Discontinued 3–12 months *N* = 50 625	Discontinued within 3 months *N* = 16 268	No use within 3 months *N* = 20 299
Age, years	74.6 (6.1)	73.8 (5.9)	74.9 (6.3)	75.3 (6.6)
Sex, female	59 808 (49%)	21 455 (42%)	7591 (47%)	8982 (44%)
Race				
White	112 775 (93%)	47 227 (93%)	15 145 (93%)	18 858 (93%)
Black	2807 (2.3%)	1217 (2.4%)	496 (3.0%)	605 (3.0%)
Other	5458 (4.5%)	2181 (4.3%)	627 (3.9%)	836 (4.1%)
Year				
2013–2015	21 791 (18%)	13 234 (26%)	3913 (24%)	2171 (11%)
2016–2019	51 434 (42%)	23 662 (47%)	7490 (46%)	9808 (48%)
2020–2022	47 815 (40%)	13 729 (27%)	4865 (30%)	8320 (41%)
Smoking	57 747 (48%)	24 476 (48%)	8257 (51%)	10 568 (52%)
CHA_2_DS_2_-VASc score	4.0 (1.5)	3.8 (1.5)	4.2 (1.5)	4.2 (1.5)
CHA_2_DS_2_-VASc score category				
1	2167 (1.8%)	2073 (4.1%)	347 (2.1%)	463 (2.3%)
2	15 624 (13%)	8644 (17%)	1932 (12%)	2302 (11%)
3	28 212 (23%)	12 553 (25%)	3379 (21%)	4296 (21%)
4	31 277 (26%)	11 883 (23%)	4004 (25%)	4959 (24%)
5	24 581 (20%)	8747 (17%)	3496 (21%)	4378 (22%)
≥6	19 179 (16%)	6725 (13%)	3110 (19%)	3901 (19%)
HAS-BLED score	2.3 (0.6)	2.2 (0.7)	2.3 (0.7)	2.4 (0.7)
Combined comorbidity score	3.3 (2.5)	3.1 (2.5)	3.7 (2.7)	3.8 (2.7)
Duration of pre-ablation OAC use^b^				
0 days	4334 (3.6%)	4507 (8.9%)	140 (0.9%)	13 413 (66%)
1–60 days	11 725 (9.7%)	9581 (19%)	3346 (21%)	2305 (11%)
61–180 days	21 805 (18%)	12 367 (24%)	3993 (25%)	2828 (14%)
181–364 days	40 135 (33%)	15 481 (31%)	7619 (47%)	1753 (8.6%)
365 days	43 041 (36%)	8689 (17%)	1170 (7.2%)	0 (0%)
Frailty category				
Non-frail	36 345 (30%)	16 372 (32%)	3894 (24%)	4636 (23%)
Pre-frail	69 396 (57%)	28 078 (55%)	9296 (57%)	11 630 (57%)
Frail	15 299 (13%)	6175 (12%)	3078 (19%)	4033 (20%)
Count of prior major bleeding				
0	114 260 (94%)	47 130 (93%)	14 601 (90%)	18 176 (90%)
1	5921 (4.9%)	3022 (6.0%)	1373 (8.4%)	1660 (8.2%)
≥2	859 (0.7%)	473 (0.9%)	294 (1.8%)	463 (2.3%)
**Comorbidities**				
Falls	3979 (3.3%)	1663 (3.3%)	784 (4.8%)	1238 (6.1%)
Fractures	8231 (6.8%)	3459 (6.8%)	1430 (8.8%)	1882 (9.3%)
Hypertension	107 603 (89%)	43 869 (87%)	14 651 (90%)	18 193 (90%)
Obesity	40 465 (33%)	15 477 (31%)	5518 (34%)	6730 (33%)
Diabetes	39 191 (32%)	15 961 (32%)	5995 (37%)	7485 (37%)
Peripheral vascular disease	13 534 (11%)	5770 (11%)	2248 (14%)	2932 (14%)
Ischaemic stroke	5694 (4.7%)	1832 (3.6%)	760 (4.7%)	1023 (5.0%)
Acute myocardial infarction	6321 (5.2%)	2584 (5.1%)	1098 (6.7%)	1470 (7.2%)
Congestive heart failure	59 502 (49%)	22 331 (44%)	8633 (53%)	10 676 (53%)
Other valvular heart disease	60 602 (50%)	24 317 (48%)	8398 (52%)	9831 (48%)
Chronic kidney disease	20 716 (17%)	8294 (16%)	3485 (21%)	4260 (21%)
Dementia	3413 (2.8%)	1328 (2.6%)	648 (4.0%)	1052 (5.2%)
COPD	25 657 (21%)	10 641 (21%)	4280 (26%)	5507 (27%)
COVID	4025 (3.3%)	882 (1.7%)	445 (2.7%)	803 (4.0%)
Cardiomyopathy	31 234 (26%)	11 134 (22%)	4426 (27%)	5476 (27%)
**Medications**				
ACE inhibitor or ARB	72 674 (60%)	28 515 (56%)	9779 (60%)	11 102 (55%)
Beta blockers	101 842 (84%)	40 528 (80%)	13 483 (83%)	15 959 (79%)
Calcium channel blockers	58 549 (48%)	23 414 (46%)	7916 (49%)	9053 (45%)
Diuretics	69 791 (58%)	26 214 (52%)	9670 (59%)	11 392 (56%)
Lipid lowering drugs	83 607 (69%)	32 881 (65%)	11 071 (68%)	12 963 (64%)
Antiarrhythmic agents	78 622 (65%)	30 903 (61%)	9867 (61%)	10 656 (52%)
Rate control agents	105 443 (87%)	42 738 (84%)	14 118 (87%)	16 639 (82%)
P2Y12 inhibitor	11 978 (9.9%)	5739 (11%)	2013 (12%)	3558 (18%)
Proton-pump inhibitors	47 343 (39%)	18 384 (36%)	6155 (38%)	7926 (39%)
Parkinsonism drugs	4254 (3.5%)	1941 (3.8%)	712 (4.4%)	928 (4.6%)
Opioids	40 845 (34%)	18 543 (37%)	6303 (39%)	7554 (37%)
**Procedures**				
Cardioversion	48 100 (40%)	17 860 (35%)	5889 (36%)	5909 (29%)
Colonoscopy	12 005 (9.9%)	5730 (11%)	1780 (11%)	2288 (11%)

OAC, oral anticoagulants; CHA_2_DS_2_-VASc, Congestive Heart failure (or left ventricular systolic dysfunction), Hypertension (blood pressure consistently >140/90 mm Hg or treated with medication), Age (≥75 y), Diabetes mellitus, Prior Stroke or transient ischaemic attack or thromboembolism, Vascular disease (e.g. peripheral artery disease, myocardial infarction, aortic plaque), age 65–74 y, Sex category (i.e. female sex); HAS-BLED, Hypertension, Abnormal renal/liver function, Stroke, Bleeding history or predisposition, Labile international normalized ratio, Elderly (>65 y), Drugs/alcohol concomitantly; COPD, chronic obstructive pulmonary disease; ACE inhibitor, angiotensin-converting enzyme inhibitor; ARB, angiotensin II receptor blocker.

^a^The continued group included 27 815 patients who were censored during the 12-month follow-up and there were 6714 (3%), and 10 154 (5%) patients censored by 2 months and 3 months after ablation, respectively; the main censoring reasons were disenrolment (76%) and death (24%).

^b^A 30-day gap was allowed between refills when calculating the duration.

Over the study period, the use of warfarin as the initial OAC decreased from 53% in 2013 to 8% in 2022, while the use of direct OACs increased over time and the most common initial OAC was apixaban followed by rivaroxaban (*Figure 2*). In 2022, 59% of patients initiated apixaban after ablation.

**Figure 2:**
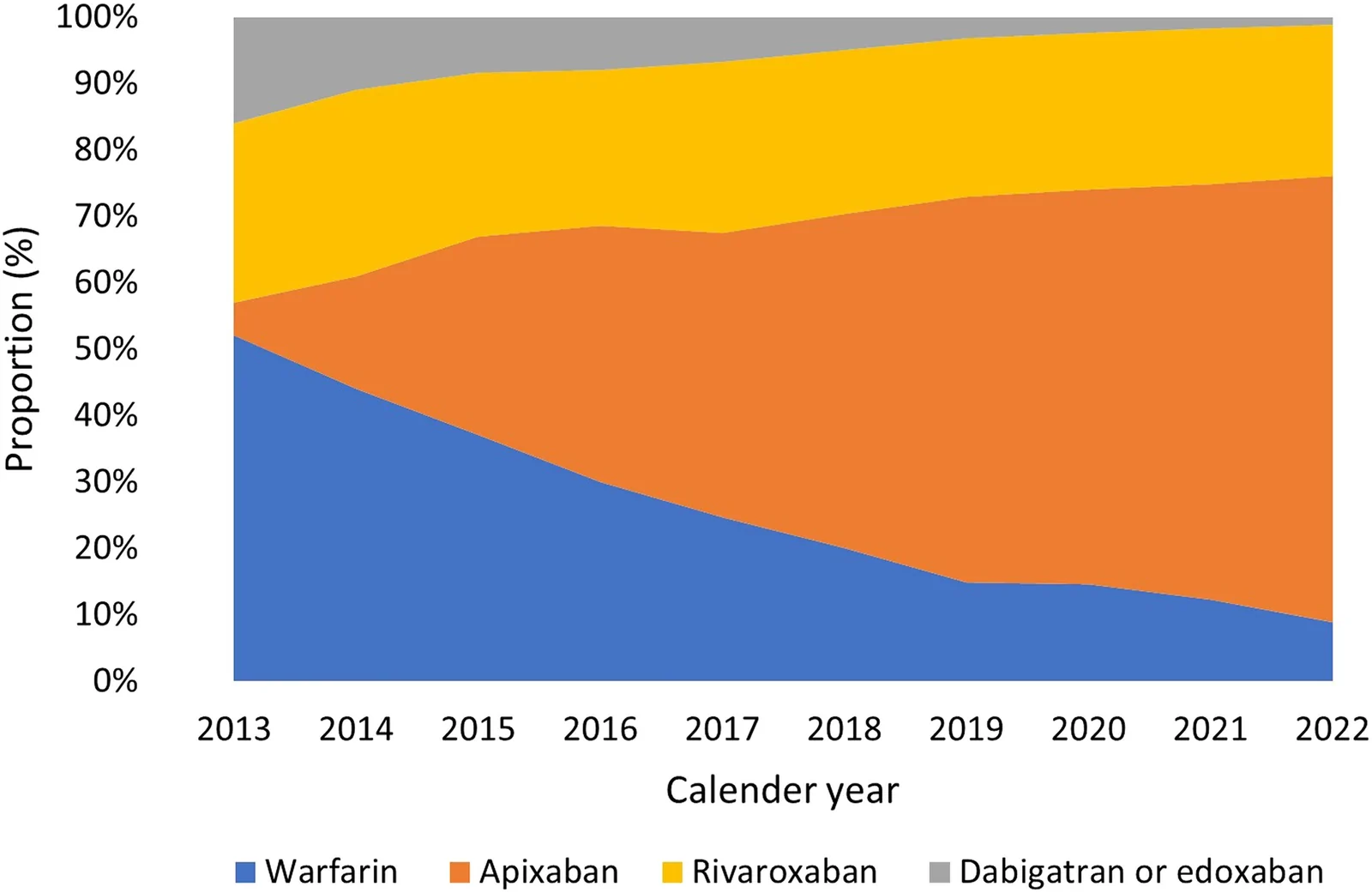
Trends of initial oral anticoagulation after ablation over the study period.

The median follow-up time was 365 days, and 78% of patients had a complete 1-year follow-up (see Supplementary material online, *Table S2*). Among patients who were retained by 12 months, 45% of patients had discontinued anticoagulation. *Figure 3* shows that discontinuation was more common in low-risk patients, e.g. the discontinuation rate at 12 months post-ablation was 61%, 49%, and 44% for CHA_2_DS_2_-VASc scores of 1, 2, and ≥3, respectively. These patterns remained consistent when stratifying by sex or age group (see Supplementary material online, *Figure S1*). In patients who discontinued within 3 months, 61% reinitiated OAC within 1-year post-ablation, while in the discontinuation 3–12 month group, 37% of patients reinitiated OAC. The overall rate of repeated ablation within 1 year was 26%; the discontinuation in 3 months group and the no-use group had a higher rate of repeated ablation (*Table 2*). We identified 3379 (1.6%), 1310 (0.6%), and 10 129 (4.9%) patients who had major bleeding, ischaemic stroke, and mortality within 1 year after ablation, respectively.

**Figure 3:**
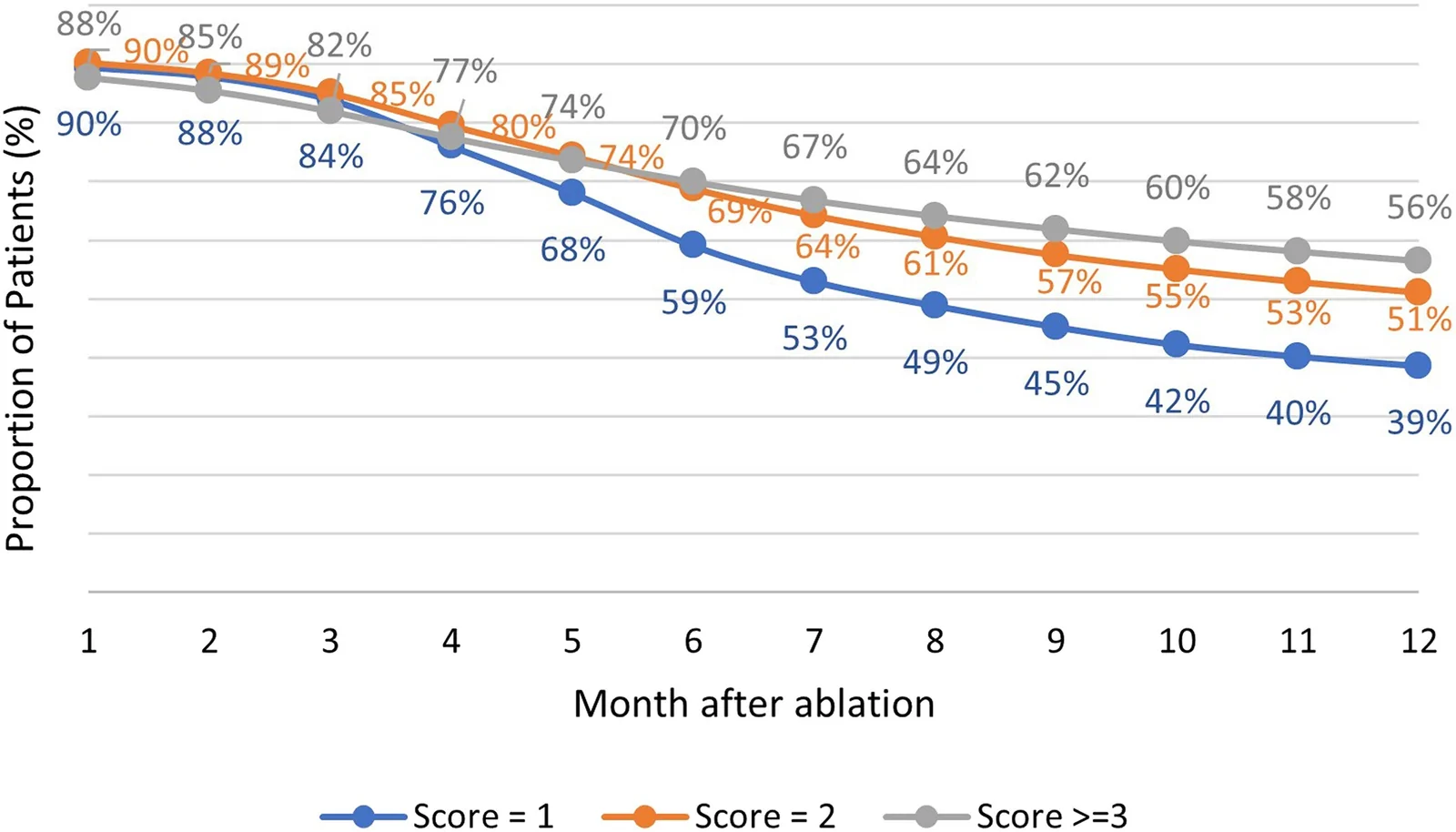
Proportion of patients remaining on anticoagulation after ablation by CHA_2_DS_2_-VASc score.

**Table 2:** Post-ablation medications and procedures

	Continued use *N* = 121 040	Discontinued 3–12 months *N* = 50 625	Discontinued within 3 months *N* = 16 268	No use within 3 months *N* = 20 299
**OAC duration, days** ^a^	365 (322, 365)	240 (156, 319)	166 (69, 307)	0 (0, 0)
**OAC reinitiation**	—	18 944 (37%)	9955 (61%)	4190 (21%)
**Time to OAC reinitiation, days** ^a,b^	—	234 (176, 296.5)	89 (58, 132)	169 (120, 245)
**Medications**				
P2Y12 inhibitors	7915 (6.5%)	6180 (12%)	1798 (11%)	2906 (14%)
Antiarrhythmic agents	63 320 (52%)	24 829 (49%)	6998 (43%)	7555 (37%)
Rate control agents	91 319 (75%)	37 553 (74%)	12 005 (74%)	13 721 (68%)
**Procedures**				
Cardioversion	21 827 (18%)	7429 (15%)	2358 (14%)	2566 (13%)
Catheter ablation	31 610 (26%)	12 896 (25%)	4674 (29%)	5773 (28%)
Surgical ablation	322 (0.3%)	210 (0.4%)	67 (0.4%)	61 (0.3%)
Left atrial appendage occlusion	1556 (1.3%)	2946 (5.8%)	534 (3.3%)	718 (3.5%)

OAC, oral anticoagulants.

^a^Data are presented as median (interquartile range [Q1, Q3]).

^b^Time since last day of supply until a new refill.

Patients who were older, female, had a higher CHA_2_DS_2_-VASc scores or underwent the procedure in more recent years were less likely to discontinue OAC after ablation (*Figure 4*). The number of prior bleedings was a particularly strong predictor of OAC discontinuation (HR [95% CI] = 1.16 [1.11–1.20] for one prior bleeding and HR = 1.25 [1.14–1.37] for two or more prior bleedings compared to no prior bleedings) while longer duration of pre-ablation OAC use were strongly associated with OAC continuation (HR = 0.48 [0.47–0.50] for a prior use of more than 180 days compared to 0–60 days). Notably, patients used warfarin at baseline were 10% more likely to discontinue compared to patients used direct OACs. A complete list of LASSO-selected predictors is shown in Supplementary material online, *Table S3*. Results remained similar when we studied OAC discontinuation at any time post-ablation (see Supplementary material online, *Table S3*). In patients with elevated stroke risk, a higher frailty score (HR = 1.79[1.49–2.15]) was the strongest predictor of discontinuation (see Supplementary material online, *Figure S2*). Using alternative discontinuation definitions based on 30-day or 90-day refill gaps yielded similar prescribing patterns (see Supplementary material online, *Tables S4*, *S5*, and Supplementary material online, *Figure S3*) and consistent predictors of treatment discontinuation (see Supplementary material online, *Tables S6* and *S7*).

**Figure 4:**
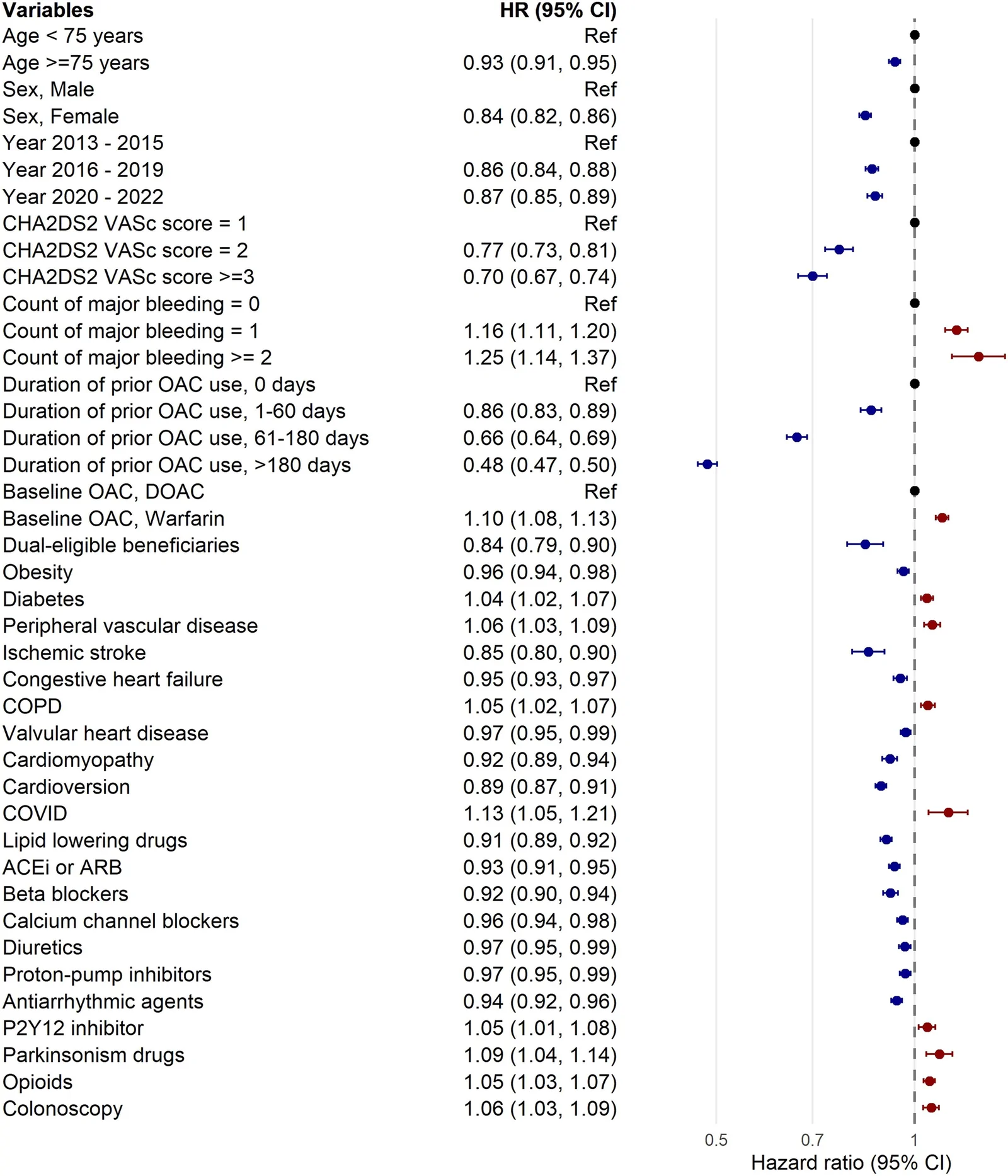
Predictors of OAC discontinuation after ablation. Hazard ratios were estimated using multivariable Cox regression, adjusting for all selected predictors. OAC, oral anticoagulants; COPD, chronic obstructive pulmonary disease; ACE inhibitor, angiotensin-converting enzyme inhibitor; ARB, angiotensin II receptor blocker.

## Discussion

In this cohort study utilizing a national US claims dataset, we assessed patterns of discontinuation of post-ablation OAC therapy in AF patients in routine practice. We found that 18% of patients discontinued or did not use OAC within the first 3 months after ablation, and 44% of patients with CHA_2_DS_2_-VASc score ≥3 discontinued OAC within 1 year after ablation. Apixaban was the most commonly used OAC agent, and the use of warfarin declined over time. The overall rate of repeated ablation was 26%. Predictors of discontinuation included prior major bleeding, lower CHA_2_DS_2_-VASc score, shorter pre-ablation OAC duration, frailty, and use of warfarin vs. direct OACs.

Contemporary data on post-ablation OAC patterns in the U.S. remain limited. We were only able to identify two previous studies exploring these patterns. In a cohort study of 6886 AF patients from a national administrative claims database (OptumLabs data warehouse) between 2005 and 2014, 39.5% of patients discontinued OAC at 3 months and 68.7% discontinued at 12 months post-procedure,^11^ higher than the observed rates in our study. However, they required the patients to have at least 1 prescription for OAC filled while our study population was not restricted. In addition, they identified a trend towards reduction in the rate of OAC discontinuation after ablation over the study period 2005 to 2014, which aligns with our finding that patients treated in more recent years were less likely to discontinue OAC after ablation, reflecting an evolving post-procedural management strategy. Another US cohort of 21 595 patients (CHA_2_DS_2_-VASc ≥2 for men and ≥3 for women) from the Outcomes Registry for Better Informed Treatment of Atrial Fibrillation (ORBIT-AF I 2010–2011 and ORBIT-AF II 2013–2016) registries reported that 23% discontinued OAC at any point post-ablation,^12^ which was lower than our findings in the subgroup with elevated CHA_2_DS_2_-VASc score. In Europe, discontinuation of OAC after ablation remains relatively common despite the 2024 ESC guidelines recommending a minimum anticoagulation of 2 months and continued stroke prevention therapy in patients at risk of stroke.^17^ For example, a multicentre registry of 1273 patients in the UK found that 64% of patients (50% patients had a CHA_2_DS_2_-VASc ≥2) stopped OAC after ablation.^18^ A survey of European electrophysiologists showed that 16% would be comfortable discontinuing OAC therapy after an apparently successful AF ablation, even in patients with a high stroke risk of CHA_2_DS_2_-VASc ≥2.^19^ These data collectively suggest considerable practice variation.

According to the 2023 ACC/AHA/HRS guideline, patients with elevated stroke risk are advised to continue OAC beyond the initial three months post-ablation. Our study revealed that the decision to discontinue at this point was likely influenced by the trade-off between the bleeding risk and stroke risk at baseline.^17,20^ Patients with a longer duration of prior OAC use were less likely to discontinue, potentially reflecting greater patient tolerability of OAC. In contrast, patients treated with warfarin were more likely to discontinue than those receiving direct OACs, consistent with a previous meta-analysis demonstrating substantially lower adherence to warfarin (50%) than to apixaban (74%) or rivaroxaban (73%).^21^ This difference likely reflects the greater monitoring burden, dietary restrictions, and bleeding concerns associated with warfarin.^22,23^ A recent systematic review suggested that younger age, multimorbidity, polypharmacy, and low income are important predictors of medication non-adherence, whereas diabetes, hypertension, and prior stroke or transient ischaemic attack have been associated with lower rates of primary non-adherence among patients with AF.^24^ Similarly, a recent retrospective study of long-term patterns of direct OAC adherence reported that lower CHA_2_DS_2_-VASc scores, impaired renal function, baseline antiarrhythmic drug use, and history of falls with injury were associated with early treatment discontinuation.^25^ Our findings were broadly consistent with these observations and further suggest that in patients at elevated risk of stroke, frailty may be an important contributing factor to discontinuing OAC. Frailty has been associated with increased risks of bleeding, stroke, and mortality after AF diagnosis,^26,27^ which may influence clinicians to discontinue anticoagulation despite ongoing thromboembolic risk. Importantly, our analysis used prescription fill data to identify treatment discontinuation and therefore cannot determine whether OAC cessation was the result of a shared medical decision following successful ablation, the occurrence of an adverse event such as bleeding, or simply poor patient adherence. Future studies incorporating richer clinical data, including reasons for treatment changes and patient preferences, are needed to better understand the determinants of OAC discontinuation after AF ablation and to guide quality improvement interventions.

The optimal OAC management following AF ablation remains uncertain, and this is largely because the impact associated with practice variation is unclear. Observational data regarding the stroke risk after catheter ablation of AF are mixed and vary across study design and methods. For example, in an analysis of 4050 patients undergoing catheter ablation with a CHA_2_DS_2_-VASc score ≥2 in Denmark, the risk of thromboembolism after ablation was low and comparable in those who did (0.93/100 patient years) and did not (0.97/100 patient years) discontinue anticoagulation > 3 months after ablation.^28^ Nevertheless, a US study of 6866 patients found that the risk of stroke was increased in patients with a CHA_2_DS_2_-VASc score ≥ 2 who discontinued OAC after 3 months (HR, 2.48 [95% CI, 1.11 to 5.52]; *P* < 0.05).^11^ Meta-analyses of observational studies have attempted to evaluate the safety of OAC discontinuation, but findings remain inconclusive.^29–32^ The recent ALONE-AF trial (Anticoagulation One year after Ablation of Atrial Fibrillation in Patients with Atrial Fibrillation) in South Korea included 840 patients from 2020 to 2023 who had no detected recurrence of atrial arrhythmia for at least 1 year after catheter ablation for AF, and found that discontinuing OAC therapy resulted in a lower risk for the composite outcome of stroke, systemic embolism, and major bleeding vs. continuing therapy (absolute difference, −1.9 percentage points [95%CI, −3.5 to −0.3]; *P* = 0.02).^33^ Similarly, the OCEAN trial (The Optimal Anticoagulation for Enhanced Risk Patients Post-Catheter Ablation for Atrial Fibrillation) found that among patients who had had successful catheter ablation at least 1 year earlier and had risk factors for stroke, treatment with rivaroxaban did not result in a significantly lower incidence of a composite of stroke, systemic embolism, or new covert embolic stroke than treatment with aspirin.^34^ These recently published trials have not yet been incorporated into AF consensus guidelines. Importantly, both OCEAN and ALONE-AF acknowledged the inclusion of patients with relatively low baseline stroke risk and low AF burden as a limitation. While these studies support the safety of OAC discontinuation after ablation among carefully selected low-risk patients with very low AF burden, the comparative benefits and risks in patients with higher AF burden and elevated stroke risk remain uncertain.

Our study is strengthened by using data from a nationwide population with comprehensive data on dispensed OAC prescriptions. This study has several limitations. First, the study population was aged ≥65 and our findings may not generalize to younger patients. Second, while claims data provide medication dispensing, it does not provide precise information on actual medication adherence or usage, which could result in misclassification of exposure. Likewise, patients without evidence of OAC dispensing during the first 3 months after ablation likely represent a heterogeneous population, including those with contraindications to anticoagulation, those who never initiated therapy, those who discontinued treatment early, and those who did not fill a prescription. Consequently, the underlying reasons for the absence of OAC dispensing could not be determined. Third, we did not have AF subtype data and rhythm monitoring data after ablation, and clinical variables such as laboratory measures were not available, and we may not fully account for all clinical indications which may have influenced prescribing decisions. Recent data indicate that pulsed field ablation may be associated with a lower thromboembolic risk compared to traditional thermal modalities.^35^ However, administrative claims data do not capture imaging findings or procedural details (e.g. ablation energy source and lesion set) and consequently we were unable to account for potential differences with various ablation techniques. Fourth, as an observational study, this analysis is inherently subject to measurement errors and residual confounding, which limit our ability to draw causal inferences. While we did not perform a formal comparative analysis of clinical outcomes across different OAC usage patterns, the descriptive nature of our study mitigates the risk that these biases would invalidate our primary observations. Further research is warranted utilizing longitudinal datasets with richer clinical details and advanced causal inference methods to examine the benefit-risk profile and clinical outcomes of continuing vs. discontinuing anticoagulation in patients undergoing AF ablation. Lastly, our study used the CHA_2_DS_2_-VASc score because it was the recommended stroke risk assessment tool in the contemporaneous U.S. AF guidelines and therefore reflects the clinical framework in which treatment decisions were made. We acknowledge that recent European guidelines have adopted the CHA_2_DS_2_-VA score, reflecting emerging evidence that female sex may act primarily as a risk modifier rather than an independent stroke risk factor.^36–38^ Although subgroup analyses by sex demonstrated similar patterns of OAC discontinuation, future studies are warranted to evaluate whether sex modifies post-ablation anticoagulation management or its clinical consequences under the CHA_2_DS_2_-VA framework.

In conclusions, utilizing a large-scale US nationwide insurance claims database, we observed high rates of OAC discontinuation following AF ablation, even among patients at high risk for stroke. This pattern deviated significantly from current guideline recommendations, which advise continued therapy regardless of procedural success in high-risk individuals. The decision of discontinuation was likely influenced by baseline bleeding risk, stroke risk, prior use of OAC, and frailty status. These findings highlight important gaps between recommended and observed practice and underscore the need for a clearer consensus and tailored strategies to optimize OAC treatment in patients undergoing catheter ablation.

## Lead author biography


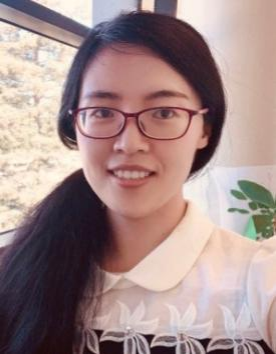
Dr. Xiaojuan Liu is a Postdoctoral Research Fellow at Brigham and Women’s Hospital and Harvard Medical School. She holds a PhD in Epidemiology and an MS in Statistics from Stanford University. Her research intersects pharmacoepidemiology, cardiovascular prevention, and ageing, with a particular focus on comparative effectiveness research using causal inference methods. She leverages real-world data, proteomics, and population-based cohorts to investigate the effects of treatments and life-course factors on cardiovascular disease and longevity. Supported by an American Heart Association Postdoctoral Fellowship, her current work investigates atrial fibrillation ablation, aiming to advance evidence-based medicine through multidisciplinary collaboration and peer-reviewed research.

## Supplementary Material

oeag128_Supplementary_Data

## Data Availability

A data use agreement is required for the data source in this study. The data use agreement does not permit the authors to share patient level source data or data derivatives with individuals and institutions not covered under the data use agreement. The database used in this study is accessible to other researchers by contacting the data providers and obtaining data use agreement or licence.
