# Supplementary material for: Discontinuation of oral anticoagulants is common after catheter ablation in older patients with atrial fibrillation
Source: Eur Heart J Open. 2026 Aug 18;6(5):oeag128. doi: 10.1093/ehjopen/oeag128 (PMC13650707; doi:10.1093/ehjopen/oeag128)
Supplement: Supplementary file 1 [file oeag128_supplementary_data.docx]

**Prescribing patterns of oral anticoagulants after catheter ablation for atrial fibrillation**

**Supplementary Tables and Figures**

**Table S1** Complete list of pre-ablation characteristics of study population by pattern groups

**Table S2** Follow-up time and censoring reasons

**Table S3** Complete list of LASSO-selected predictors associated with anticoagulation discontinuation after ablation

**Table S4** Pre-ablation characteristics of study population by pattern groups with anticoagulation discontinuation defined by alternative refill gaps

**Table S5** Post-ablation medications and procedures by pattern groups with anticoagulation discontinuation defined by alternative refill gaps

**Table S6** Sensitivity analysis of LASSO-selected predictors associated with anticoagulation discontinuation after ablation with discontinuation defined by a 30-day refill gap

**Table S7** Sensitivity analysis of LASSO-selected predictors associated with anticoagulation discontinuation after ablation with discontinuation defined by a 90-day refill gap

**Figure S1** Proportion of patients remaining on anticoagulation after ablation in subgroups by age and sex

**Figure S2** Predictors of OAC discontinuation 3 –12 months after ablation in patients with elevated stroke risk (CHA_2_DS_2_-VASc score ≥ 3 for men or ≥ 2 for women).

**Figure S3**. Proportion of patients remaining on anticoagulation after ablation by CHA_2_DS_2_-VASc score. Left panel used discontinuation defined by a 30-day gap and right panel used discontinuation defined by a 60-day gap.

**Supplementary Method**

**Table S1**. Complete list of pre-ablation characteristics of study population by pattern groups

| **Characteristics** | **Continued  N = 121,040** | **Discontinued 90 days – 12 months  N = 50,625** | **Discontinued within 90 days  N = 16,268** | **No use within 90 days  N = 20,299** | **Overall**  **N = 208,232** |
| --- | --- | --- | --- | --- | --- |
| Age, years | 74.6 (6.1) | 73.8 (5.9) | 74.9 (6.3) | 75.3 (6.6) | 74.5 (6.1) |
| Age category |  |  |  |  |  |
| < 75 years | 66,282 (55%) | 30,608 (60%) | 8,509 (52%) | 10,402 (51%) | 115,801 (56%) |
| ≥ 75 years | 54,758 (45%) | 20,017 (40%) | 7,759 (48%) | 9,897 (49%) | 92,431 (44%) |
| Sex, female | 59,808 (49%) | 21,455 (42%) | 7,591 (47%) | 8,982 (44%) | 97,836 (47%) |
| Race |  |  |  |  |  |
| White | 112,775 (93%) | 47,227 (93%) | 15,145 (93%) | 18,858 (93%) | 194,005 (93%) |
| Black | 2,807 (2.3%) | 1,217 (2.4%) | 496 (3.0%) | 605 (3.0%) | 5,125 (2.5%) |
| Other | 5,458 (4.5%) | 2,181 (4.3%) | 627 (3.9%) | 836 (4.1%) | 9,102 (4.4%) |
| Region |  |  |  |  |  |
| Northeast | 21,343 (18%) | 7,652 (15%) | 2,302 (14%) | 2,540 (13%) | 33,837 (16%) |
| Midwest | 29,885 (25%) | 11,268 (22%) | 3,801 (23%) | 4,768 (23%) | 49,722 (24%) |
| South/Other | 47,967 (40%) | 21,207 (42%) | 7,191 (44%) | 9,332 (46%) | 85,697 (41%) |
| West | 21,845 (18%) | 10,498 (21%) | 2,974 (18%) | 3,659 (18%) | 38,976 (19%) |
| Year |  |  |  |  |  |
| 2013 - 2015 | 21,791 (18%) | 13,234 (26%) | 3,913 (24%) | 2,171 (11%) | 41,109 (20%) |
| 2016 - 2019 | 51,434 (42%) | 23,662 (47%) | 7,490 (46%) | 9,808 (48%) | 92,394 (44%) |
| 2020 - 2022 | 47,815 (40%) | 13,729 (27%) | 4,865 (30%) | 8,320 (41%) | 74,729 (36%) |
| Dual-eligible beneficiaries | 3,285 (2.7%) | 1,030 (2.0%) | 422 (2.6%) | 488 (2.4%) | 5,225 (2.5%) |
| Smoking | 57,747 (48%) | 24,476 (48%) | 8,257 (51%) | 10,568 (52%) | 101,048 (49%) |
| Alcohol abuse or dependence | 2,231 (1.8%) | 1,104 (2.2%) | 358 (2.2%) | 487 (2.4%) | 4,180 (2.0%) |
| Obesity | 40,465 (33%) | 15,477 (31%) | 5,518 (34%) | 6,730 (33%) | 68,190 (33%) |
| CHA_2_DS_2_-VASc score | 4.0 (1.5) | 3.8 (1.5) | 4.2 (1.5) | 4.2 (1.5) | 4.0 (1.5) |
| CHA_2_DS_2_-VASc score category |  |  |  |  |  |
| 1 | 2,167 (1.8%) | 2,073 (4.1%) | 347 (2.1%) | 463 (2.3%) | 5,050 (2.4%) |
| 2 | 15,624 (13%) | 8,644 (17%) | 1,932 (12%) | 2,302 (11%) | 28,502 (14%) |
| 3 | 28,212 (23%) | 12,553 (25%) | 3,379 (21%) | 4,296 (21%) | 48,440 (23%) |
| 4 | 31,277 (26%) | 11,883 (23%) | 4,004 (25%) | 4,959 (24%) | 52,123 (25%) |
| 5 | 24,581 (20%) | 8,747 (17%) | 3,496 (21%) | 4,378 (22%) | 41,202 (20%) |
| >=6 | 19,179 (16%) | 6,725 (13%) | 3,110 (19%) | 3,901 (19%) | 32,915 (16%) |
| HAS-BLED score | 2.3 (0.6) | 2.2 (0.7) | 2.3 (0.7) | 2.4 (0.7) | 2.3 (0.7) |
| HAS-BLED score category |  |  |  |  |  |
| <3 | 84,947 (70%) | 35,759 (71%) | 10,435 (64%) | 12,699 (63%) | 143,840 (69%) |
| 3 | 32,232 (27%) | 13,266 (26%) | 5,155 (32%) | 6,622 (33%) | 57,275 (28%) |
| >=4 | 3,861 (3.2%) | 1,600 (3.2%) | 678 (4.2%) | 978 (4.8%) | 7,117 (3.4%) |
| Combined comorbidity score | 3.3 (2.5) | 3.1 (2.5) | 3.7 (2.7) | 3.8 (2.7) | 3.3 (2.5) |
| Duration of prior OAC use, days | 248.0 (126.9) | 179.8 (132.6) | 193.3 (120.8) | 40.4 (74.5) | 206.9 (138.5) |
| Duration of prior OAC use category |  |  |  |  |  |
| 0 days | 4,334 (3.6%) | 4,507 (8.9%) | 140 (0.9%) | 13,413 (66%) | 22,394 (11%) |
| 1-60 days | 11,725 (9.7%) | 9,581 (19%) | 3,346 (21%) | 2,305 (11%) | 26,957 (13%) |
| 61-180 days | 21,805 (18%) | 12,367 (24%) | 3,993 (25%) | 2,828 (14%) | 40,993 (20%) |
| 181-364 days | 40,135 (33%) | 15,481 (31%) | 7,619 (47%) | 1,753 (8.6%) | 64,988 (31%) |
| 365 days | 43,041 (36%) | 8,689 (17%) | 1,170 (7.2%) | 0 (0%) | 52,900 (25%) |
| **Comorbidities** |  |  |  |  |  |
| Frailty category |  |  |  |  |  |
| Non-frail | 36,345 (30%) | 16,372 (32%) | 3,894 (24%) | 4,636 (23%) | 61,247 (29%) |
| Pre-frail | 69,396 (57%) | 28,078 (55%) | 9,296 (57%) | 11,630 (57%) | 118,400 (57%) |
| Frail | 15,299 (13%) | 6,175 (12%) | 3,078 (19%) | 4,033 (20%) | 28,585 (14%) |
| Count of major bleeding |  |  |  |  |  |
| 0 | 114,260 (94%) | 47,130 (93%) | 14,601 (90%) | 18,176 (90%) | 194,167 (93%) |
| 1 | 5,921 (4.9%) | 3,022 (6.0%) | 1,373 (8.4%) | 1,660 (8.2%) | 11,976 (5.8%) |
| >=2 | 859 (0.7%) | 473 (0.9%) | 294 (1.8%) | 463 (2.3%) | 2,089 (1.0%) |
| Falls | 3,979 (3.3%) | 1,663 (3.3%) | 784 (4.8%) | 1,238 (6.1%) | 7,664 (3.7%) |
| Fractures | 8,231 (6.8%) | 3,459 (6.8%) | 1,430 (8.8%) | 1,882 (9.3%) | 15,002 (7.2%) |
| Hypertension | 107,603 (89%) | 43,869 (87%) | 14,651 (90%) | 18,193 (90%) | 184,316 (89%) |
| Hyperlipidemia | 98,211 (81%) | 40,434 (80%) | 13,412 (82%) | 16,475 (81%) | 168,532 (81%) |
| Diabetes | 39,191 (32%) | 15,961 (32%) | 5,995 (37%) | 7,485 (37%) | 68,632 (33%) |
| Cerebrovascular disease | 23,270 (19%) | 9,576 (19%) | 3,556 (22%) | 4,493 (22%) | 40,895 (20%) |
| Acute myocardial infarction | 6,321 (5.2%) | 2,584 (5.1%) | 1,098 (6.7%) | 1,470 (7.2%) | 11,473 (5.5%) |
| Transient ischemic attack | 4,801 (4.0%) | 1,779 (3.5%) | 691 (4.2%) | 785 (3.9%) | 8,056 (3.9%) |
| Ischemic stroke | 5,694 (4.7%) | 1,832 (3.6%) | 760 (4.7%) | 1,023 (5.0%) | 9,309 (4.5%) |
| Ischemic heart disease | 66,526 (55%) | 27,448 (54%) | 9,634 (59%) | 12,660 (62%) | 116,268 (56%) |
| Congestive heart failure | 59,502 (49%) | 22,331 (44%) | 8,633 (53%) | 10,676 (53%) | 101,142 (49%) |
| Peripheral vascular disease | 13,534 (11%) | 5,770 (11%) | 2,248 (14%) | 2,932 (14%) | 24,484 (12%) |
| Non-ischemic stroke | 3,174 (2.6%) | 1,147 (2.3%) | 439 (2.7%) | 475 (2.3%) | 5,235 (2.5%) |
| Acute renal failure | 16,327 (13%) | 6,507 (13%) | 3,006 (18%) | 3,716 (18%) | 29,556 (14%) |
| Chronic kidney disease | 20,716 (17%) | 8,294 (16%) | 3,485 (21%) | 4,260 (21%) | 36,755 (18%) |
| Liver disease | 7,932 (6.6%) | 3,278 (6.5%) | 1,106 (6.8%) | 1,405 (6.9%) | 13,721 (6.6%) |
| Dementia | 3,413 (2.8%) | 1,328 (2.6%) | 648 (4.0%) | 1,052 (5.2%) | 6,441 (3.1%) |
| COPD | 25,657 (21%) | 10,641 (21%) | 4,280 (26%) | 5,507 (27%) | 46,085 (22%) |
| Anemia | 29,401 (24%) | 12,724 (25%) | 4,940 (30%) | 6,528 (32%) | 53,593 (26%) |
| Valvular heart disease | 60,602 (50%) | 24,317 (48%) | 8,398 (52%) | 9,831 (48%) | 103,148 (50%) |
| Arterial embolism | 289 (0.2%) | 103 (0.2%) | 46 (0.3%) | 66 (0.3%) | 504 (0.2%) |
| Post-thrombotic syndrome | 78 (<0.1%) | 25 (<0.1%) | 14 (<0.1%) | 10 (<0.1%) | 127 (<0.1%) |
| Cardiomyopathy | 31,234 (26%) | 11,134 (22%) | 4,426 (27%) | 5,476 (27%) | 52,270 (25%) |
| Cardioversion | 48,100 (40%) | 17,860 (35%) | 5,889 (36%) | 5,909 (29%) | 77,758 (37%) |
| Coronary revascularization | 3,184 (2.6%) | 1,638 (3.2%) | 603 (3.7%) | 882 (4.3%) | 6,307 (3.0%) |
| Osteoarthritis | 46,046 (38%) | 18,905 (37%) | 6,579 (40%) | 8,447 (42%) | 79,977 (38%) |
| Coagulation defect | 2,465 (2.0%) | 1,018 (2.0%) | 449 (2.8%) | 484 (2.4%) | 4,416 (2.1%) |
| Syncope | 14,744 (12%) | 6,096 (12%) | 2,230 (14%) | 3,132 (15%) | 26,202 (13%) |
| Peptic ulcer | 3,260 (2.7%) | 1,569 (3.1%) | 588 (3.6%) | 938 (4.6%) | 6,355 (3.1%) |
| Anticoagulants, injectable | 5,063 (4.2%) | 2,331 (4.6%) | 742 (4.6%) | 442 (2.2%) | 8,578 (4.1%) |
| Lipid lowering drugs | 83,607 (69%) | 32,881 (65%) | 11,071 (68%) | 12,963 (64%) | 140,522 (67%) |
| Fibrates | 4,882 (4.0%) | 2,077 (4.1%) | 669 (4.1%) | 705 (3.5%) | 8,333 (4.0%) |
| Nitrates | 14,127 (12%) | 5,813 (11%) | 2,250 (14%) | 2,943 (14%) | 25,133 (12%) |
| Statins | 80,005 (66%) | 31,235 (62%) | 10,482 (64%) | 12,125 (60%) | 133,847 (64%) |
| Antihypertensive medication | 109,104 (90%) | 43,910 (87%) | 14,633 (90%) | 17,517 (86%) | 185,164 (89%) |
| ACE inhibitors | 39,610 (33%) | 16,395 (32%) | 5,706 (35%) | 6,113 (30%) | 67,824 (33%) |
| ARBs | 36,941 (31%) | 13,823 (27%) | 4,629 (28%) | 5,604 (28%) | 60,997 (29%) |
| Beta blockers | 101,842 (84%) | 40,528 (80%) | 13,483 (83%) | 15,959 (79%) | 171,812 (83%) |
| Calcium channel blockers | 58,549 (48%) | 23,414 (46%) | 7,916 (49%) | 9,053 (45%) | 98,932 (48%) |
| Diuretics | 69,791 (58%) | 26,214 (52%) | 9,670 (59%) | 11,392 (56%) | 117,067 (56%) |
| Loop diuretics | 48,114 (40%) | 17,349 (34%) | 7,006 (43%) | 8,206 (40%) | 80,675 (39%) |
| Thiazide and thiazide-like diuretics | 18,656 (15%) | 7,346 (15%) | 2,517 (15%) | 2,909 (14%) | 31,428 (15%) |
| Glucose lowering agents | 26,406 (22%) | 10,543 (21%) | 3,982 (24%) | 4,623 (23%) | 45,554 (22%) |
| Insulin | 8,141 (6.7%) | 3,132 (6.2%) | 1,298 (8.0%) | 1,350 (6.7%) | 13,921 (6.7%) |
| Metformin | 19,108 (16%) | 7,518 (15%) | 2,714 (17%) | 3,157 (16%) | 32,497 (16%) |
| Sulfonylurea | 8,631 (7.1%) | 3,780 (7.5%) | 1,436 (8.8%) | 1,717 (8.5%) | 15,564 (7.5%) |
| Other oral hypoglycemic agents | 5,159 (4.3%) | 1,992 (3.9%) | 749 (4.6%) | 769 (3.8%) | 8,669 (4.2%) |
| Antidepressants | 48,599 (40%) | 20,541 (41%) | 7,045 (43%) | 8,721 (43%) | 84,906 (41%) |
| SSRI/SNRI antidepressants | 25,480 (21%) | 10,275 (20%) | 3,754 (23%) | 4,660 (23%) | 44,169 (21%) |
| Tricyclic antidepressants | 3,130 (2.6%) | 1,396 (2.8%) | 509 (3.1%) | 620 (3.1%) | 5,655 (2.7%) |
| Other antidepressants | 10,614 (8.8%) | 4,368 (8.6%) | 1,590 (9.8%) | 2,018 (9.9%) | 18,590 (8.9%) |
| Benzodiazepines | 21,041 (17%) | 9,335 (18%) | 3,076 (19%) | 3,842 (19%) | 37,294 (18%) |
| Anxiolytics (except benzodiazepine) | 1,875 (1.5%) | 691 (1.4%) | 270 (1.7%) | 381 (1.9%) | 3,217 (1.5%) |
| Antiarrhythmic agents | 78,622 (65%) | 30,903 (61%) | 9,867 (61%) | 10,656 (52%) | 130,048 (62%) |
| Rate control agents | 105,443 (87%) | 42,738 (84%) | 14,118 (87%) | 16,639 (82%) | 178,938 (86%) |
| Estrogen | 5,398 (4.5%) | 2,004 (4.0%) | 608 (3.7%) | 651 (3.2%) | 8,661 (4.2%) |
| Thyroid hormone replacement | 29,053 (24%) | 11,213 (22%) | 3,975 (24%) | 4,875 (24%) | 49,116 (24%) |
| H2 blockers | 9,292 (7.7%) | 3,610 (7.1%) | 1,270 (7.8%) | 1,618 (8.0%) | 15,790 (7.6%) |
| Proton-pump inhibitors | 47,343 (39%) | 18,384 (36%) | 6,155 (38%) | 7,926 (39%) | 79,808 (38%) |
| Anticonvulsants | 19,469 (16%) | 8,066 (16%) | 2,919 (18%) | 3,835 (19%) | 34,289 (16%) |
| Dementia drugs | 2,394 (2.0%) | 942 (1.9%) | 372 (2.3%) | 592 (2.9%) | 4,300 (2.1%) |
| Parkinsonism drugs | 4,254 (3.5%) | 1,941 (3.8%) | 712 (4.4%) | 928 (4.6%) | 7,835 (3.8%) |
| NSAIDs | 14,248 (12%) | 7,098 (14%) | 2,100 (13%) | 2,672 (13%) | 26,118 (13%) |
| Opioids | 40,845 (34%) | 18,543 (37%) | 6,303 (39%) | 7,554 (37%) | 73,245 (35%) |
| Antibiotics | 76,386 (63%) | 32,403 (64%) | 10,685 (66%) | 13,124 (65%) | 132,598 (64%) |
| Antiplatelet agent | 614 (0.5%) | 299 (0.6%) | 98 (0.6%) | 128 (0.6%) | 1,139 (0.5%) |
| P2Y12 inhibitor | 11,978 (9.9%) | 5,739 (11%) | 2,013 (12%) | 3,558 (18%) | 23,288 (11%) |
| Antipsychotic agents | 1,963 (1.6%) | 849 (1.7%) | 335 (2.1%) | 399 (2.0%) | 3,546 (1.7%) |
| Bronchodilators | 22,749 (19%) | 8,889 (18%) | 3,199 (20%) | 3,439 (17%) | 38,276 (18%) |
| Corticosteroids, oral | 42,876 (35%) | 18,079 (36%) | 6,187 (38%) | 7,628 (38%) | 74,770 (36%) |
| Corticosteroids, inhaled | 27,270 (23%) | 11,082 (22%) | 3,599 (22%) | 3,995 (20%) | 45,946 (22%) |
| Endoscopy | 2,067 (1.7%) | 1,163 (2.3%) | 609 (3.7%) | 749 (3.7%) | 4,588 (2.2%) |
| Colonoscopy | 12,005 (9.9%) | 5,730 (11%) | 1,780 (11%) | 2,288 (11%) | 0.6 (0.1) |
| TTR score | 0.6 (0.1) | 0.6 (0.1) | 0.6 (0.1) | 0.6 (0.1) | 8,761 (4.2%) |
| Home oxygen use | 4,835 (4.0%) | 1,806 (3.6%) | 944 (5.8%) | 1,176 (5.8%) | 98,163 (47%) |
| Emergency department visit | 56,350 (47%) | 23,556 (47%) | 8,096 (50%) | 10,161 (50%) | 113,870 (55%) |
| Hospitalization | 64,519 (53%) | 27,387 (54%) | 9,982 (61%) | 11,982 (59%) | 4.2 (7.9) |
| Number of hospital days | 4.0 (7.5) | 3.9 (7.6) | 5.6 (9.8) | 5.1 (9.4) | 9,102 (4.4%) |
| Skilled nursing home | 4,702 (3.9%) | 1,853 (3.7%) | 1,208 (7.4%) | 1,339 (6.6%) | 1.4 (10.2) |
| Number of skilled nursing home days | 1.2 (9.4) | 1.1 (8.7) | 2.6 (13.9) | 2.3 (13.6) | 16.0 (9.2) |
| Number of Office visits | 15.8 (9.0) | 16.0 (9.4) | 16.7 (9.6) | 16.3 (9.6) | 29,292 (14%) |
| Home health day | 16,181 (13%) | 6,454 (13%) | 2,963 (18%) | 3,694 (18%) | 24.9 (16.9) |
| Number of cardiologist visits | 24.7 (16.6) | 24.9 (17.4) | 26.4 (18.0) | 24.2 (16.9) | 21,803 (10%) |
| CT thoracic ventilation scan | 9,249 (7.6%) | 3,753 (7.4%) | 1,312 (8.1%) | 1,772 (8.7%) | 16,086 (7.7%) |
| Duplex scan | 5,533 (4.6%) | 2,342 (4.6%) | 840 (5.2%) | 1,017 (5.0%) | 9,732 (4.7%) |
| D-dimer test | 7,674 (6.3%) | 3,291 (6.5%) | 1,148 (7.1%) | 1,482 (7.3%) | 13,595 (6.5%) |
| Coronavirus disease of 2019 | 4,025 (3.3%) | 882 (1.7%) | 445 (2.7%) | 803 (4.0%) | 6,155 (3.0%) |

Abbreviations: OAC= oral anticoagulants; CHA_2_DS_2_-VASc = Congestive Heart failure (or left ventricular systolic dysfunction), Hypertension (blood pressure consistently >140/90 mm Hg or treated with medication), Age (≥75 y), Diabetes mellitus, Prior Stroke or transient ischemic attack or thromboembolism, Vascular disease (e.g., peripheral artery disease, myocardial infarction, aortic plaque), Age 65-74 y, Sex category (i.e., female sex); HAS-BLED = Hypertension, Abnormal renal/liver function, Stroke, Bleeding history or predisposition, Labile international normalized ratio, Elderly (>65 y), Drugs/alcohol concomitantly; COPD=chronic obstructive pulmonary disease; ACE inhibitor = Angiotensin-Converting Enzyme inhibitor; NSAIDs = nonsteroidal anti-inflammatory drugs.

**Table S2.** Follow-up time and censoring reasons

|  | **Overall**  **N = 208,232** | **Continued  N = 121,040** | **Discontinued 90 days – 12 months  N = 50,625** | **Discontinued within 90 days  N = 16,268** | **No use within 90 days  N = 20,299** |
| --- | --- | --- | --- | --- | --- |
| Follow-up, days | 365 (365, 365) | 365 (341, 365) | 365 (365, 365) | 365 (365, 365) | 365 (313, 365) |
| Censoring reasons (%) |  |  |  |  |  |
| End of patient enrollment | 36,359 (17%) | 25,508 (21%) | 4,453 (8.8%) | 2,271 (14%) | 4,127 (20%) |
| Maximum follow-up time | 161,775 (78%) | 89,358 (74%) | 44,852 (89%) | 12,984 (80%) | 14,581 (72%) |
| Death | 10,098 (4.8%) | 6,174 (5.1%) | 1,320 (2.6%) | 1,013 (6.2%) | 1,591 (7.8%) |

**Table S3**. Complete list of LASSO-selected predictors associated with anticoagulation discontinuation after ablation

|  | **Hazard ratios (95% CI)** | |
| --- | --- | --- |
|  | **Discontinued 3 – 12 months** | **Any Discontinuation** |
| Age < 75 years | Ref | Ref |
| Age >=75 years | **0.93 (0.91, 0.95)** | **0.93 (0.91, 0.95)** |
| Sex, male | Ref | Ref |
| Sex, female | **0.84 (0.82, 0.86)** | **0.87 (0.86, 0.89)** |
| Race, White | Ref | Ref |
| Race, Black | **1.10 (1.04, 1.17)** | **1.10 (1.05, 1.16)** |
| Race, Other | **0.95 (0.91, 0.99)** | **0.95 (0.91, 0.98)** |
| Region, East | Ref | Ref |
| Region, Midwest | **1.03 (1.00, 1.06)** | **1.06 (1.03, 1.08)** |
| Region, South/Other | **1.15 (1.12, 1.18)** | **1.17 (1.15, 1.20)** |
| Region, West | **1.22 (1.18, 1.26)** | **1.20 (1.16, 1.23)** |
| Year 2013 - 2015 | Ref | Ref |
| Year 2016 - 2019 | **0.86 (0.84, 0.88)** | **0.88 (0.87, 0.90)** |
| Year 2020 - 2022 | **0.87 (0.85, 0.89)** | **0.88 (0.86, 0.90)** |
| CHA_2_DS_2_-VASc score = 1 | Ref | Ref |
| CHA_2_DS_2_-VASc score3 = 2 | **0.77 (0.73, 0.81)** | **0.80 (0.76, 0.83)** |
| CHA_2_DS_2_-VASc score3 >= 3 | **0.70 (0.67, 0.74)** | **0.73 (0.69, 0.76)** |
| Count of prior major bleeding = 0 | Ref | Ref |
| Count of prior major bleeding = 1 | **1.16 (1.11, 1.20)** | **1.19 (1.15, 1.23)** |
| Count of prior major bleeding >= 2 | **1.25 (1.14, 1.37)** | **1.35 (1.25, 1.45)** |
| Duration of pre-ablation OAC use, 0 days | Ref | **Ref** |
| Duration of pre-ablation OAC use, 1-60 days | **0.86 (0.83, 0.89)** | **1.14 (1.10, 1.18)** |
| Duration of pre-ablation OAC use, 61-180 days | **0.66 (0.64, 0.69)** | **0.89 (0.86, 0.92)** |
| Duration of pre-ablation OAC use, >180 days | **0.48 (0.47, 0.50)** | **0.74 (0.71, 0.76)** |
| Baseline OAC, DOAC | **Ref** | **Ref** |
| Baseline OAC, Warfarin | **1.10 (1.08, 1.13)** | **1.07 (1.05, 1.09)** |
| Dual-eligible beneficiaries | **0.84 (0.79, 0.90)** | **0.85 (0.81, 0.90)** |
| Alcohol abuse or dependence | **1.07 (1.00, 1.15)** | Not selected |
| Obesity | **0.96 (0.94, 0.98)** | **0.98 (0.96, 0.99)** |
| Falls | **1.09 (1.03, 1.14)** | **1.12 (1.07, 1.16)** |
| Diabetes | **1.04 (1.02, 1.07)** | **1.05 (1.03, 1.07)** |
| Peripheral vascular disease | **1.06 (1.03, 1.09)** | **1.06 (1.04, 1.09)** |
| Acute myocardial infarction | **0.94 (0.90, 0.98)** | **0.94 (0.90, 0.98)** |
| Transient ischemic attack | **0.94 (0.89, 0.99)** | 0.96 (0.91, 1.01) |
| Ischemic stroke | **0.85 (0.80, 0.90)** | **0.88 (0.84, 0.92)** |
| Congestive heart failure | **0.95 (0.93, 0.97)** | **0.97 (0.95, 0.99)** |
| Non-ischemic stroke | **0.90 (0.84, 0.97)** | **0.91 (0.86, 0.96)** |
| Chronic obstructive pulmonary disease | **1.05 (1.02, 1.07)** | **1.07 (1.05, 1.09)** |
| Anemia | **1.07 (1.02, 1.11)** | **1.06 (1.04, 1.08)** |
| Valvular heart disease | **0.97 (0.95, 0.99)** | **0.98 (0.96, 0.99)** |
| Acute renal failure | **1.04 (1.01, 1.07)** | **1.07 (1.04, 1.09)** |
| Cardiomyopathy | **0.92 (0.89, 0.94)** | **0.95 (0.93, 0.97)** |
| Cardioversion | **0.89 (0.87, 0.91)** | **0.89 (0.88, 0.90)** |
| Peptic ulcer | **1.07 (1.02, 1.13)** | **1.06 (1.01, 1.11)** |
| Coronavirus disease of 2019 | **1.13 (1.05, 1.21)** | **1.12 (1.06, 1.19)** |
| Lipid lowering drugs | **0.91 (0.89, 0.92)** | **0.92 (0.90, 0.93)** |
| ACEi or ARB | **0.93 (0.91, 0.95)** | **0.95 (0.93, 0.96)** |
| Beta blockers | **0.92 (0.90, 0.94)** | **0.93 (0.91, 0.95)** |
| Calcium channel blockers | **0.96 (0.94, 0.98)** | **0.96 (0.95, 0.98)** |
| Diuretics | **0.97 (0.95, 0.99)** | **0.98 (0.96, 0.99)** |
| Estrogen | **0.93 (0.89, 0.98)** | **0.93 (0.89, 0.96)** |
| Proton-pump inhibitors | **0.97 (0.95, 0.99)** | **0.95 (0.93, 0.98)** |
| Antiarrhythmic agents | **0.94 (0.92, 0.96)** | **0.94 (0.93, 0.96)** |
| P2Y12 inhibitor | **1.05 (1.01, 1.08)** | **1.05 (1.01, 1.08)** |
| Antidepressants | **1.03 (1.01, 1.06)** | **1.04 (1.02, 1.06)** |
| Parkinsonism drugs | **1.09 (1.04, 1.14)** | **1.07 (1.03, 1.11)** |
| Opioids | **1.05 (1.03, 1.07)** | **1.05 (1.03, 1.06)** |
| Antibiotics | **1.02 (1.00, 1.04)** | **1.02 (1.00, 1.04)** |
| Bronchodilators | **0.96 (0.93, 0.98)** | **0.96 (0.93, 0.98)** |
| Corticosteroids, oral | **1.03 (1.01, 1.05)** | **1.03 (1.02, 1.05)** |
| Corticosteroids, inhaled | **0.98 (0.95, 1.00)** | **0.96 (0.94, 0.98)** |
| Colonoscopy | **1.06 (1.03, 1.09)** | **1.06 (1.03, 1.08)** |
| Coagulation defect | 1.06 (1.00, 1.13) | **1.08 (1.02, 1.14)** |
| Benzodiazepines | 1.03 (1.00, 1.05) | Not selected |
| Anticonvulsants | 1.02 (0.99, 1.04) | Not selected |
| NSAIDs | 1.02 (0.99, 1.05) | Not selected |
| Rate control agents | 0.97 (0.95, 1.00) | Not selected |
| HAS-BLED score < 3 | Ref | Not selected |
| HAS-BLED score >=4 | 1.00 (0.96, 1.04) | Not selected |
| HAS-BLED score >=4 | 1.06 (0.98, 1.14) | Not selected |
| Frailty score | Not selected | **1.82 (1.51, 2.20)** |
| Skilled nursing home | Not selected | 1.03 (0.99, 1.07) |

**Notes**: Predictors were selected by least absolute shrinkage and selection operator (LASSO) Cox regression. Hazard ratios were estimated using multivariable Cox regression, adjusting for all selected predictors. Statistically significant results are indicated in bold.

**Table S4**. Pre-ablation characteristics by pattern groups using anticoagulation discontinuation defined by alternative refill gaps

|  | **30-day gaps** | | | **90-day gaps** | | |
| --- | --- | --- | --- | --- | --- | --- |
| **Characteristics** | **Continued use  N = 92,447** | **Discontinued 3 – 12 months  N = 57,956** | **Discontinued within 3 months  N = 35,413** | **Continued use N = 135,740** | **Discontinued 3 – 12 months  N = 44,796** | **Discontinued within 3 months  N = 5,280** |
| Age, years | 74.5 (6.1) | 74.0 (5.9) | 74.8 (6.3) | 74.6 (6.1) | 73.6 (5.8) | 75.1 (6.2) |
| Sex, female | 45,705 (49%) | 25,962 (45%) | 16,194 (46%) | 67,004 (49%) | 18,300 (41%) | 2,557 (48%) |
| Race |  |  |  |  |  |  |
| White | 86,127 (93%) | 54,144 (93%) | 32,891 (93%) | 126,460 (93%) | 41,768 (93%) | 4,934 (93%) |
| Black | 2,059 (2.2%) | 1,316 (2.3%) | 1,087 (3.1%) | 3,231 (2.4%) | 1,083 (2.4%) | 148 (2.8%) |
| Other | 4,261 (4.6%) | 2,496 (4.3%) | 1,435 (4.1%) | 6,049 (4.5%) | 1,945 (4.3%) | 198 (3.8%) |
| Year |  |  |  |  |  |  |
| 2013 - 2015 | 15,174 (16%) | 14,161 (24%) | 9,190 (26%) | 25,275 (19%) | 12,023 (27%) | 1,227 (23%) |
| 2016 - 2019 | 38,209 (41%) | 27,291 (47%) | 16,160 (46%) | 58,197 (43%) | 20,980 (47%) | 2,483 (47%) |
| 2020 - 2022 | 39,064 (42%) | 16,504 (28%) | 10,063 (28%) | 52,268 (39%) | 11,793 (26%) | 1,570 (30%) |
| Smoking | 43,875 (47%) | 27,917 (48%) | 17,544 (50%) | 64,834 (48%) | 21,866 (49%) | 2,636 (50%) |
| CHA_2_DS_2_-VASc score | 4.0 (1.5) | 3.9 (1.5) | 4.1 (1.5) | 4.1 (1.5) | 3.7 (1.5) | 4.2 (1.5) |
| CHA_2_DS_2_-VASc score category |  |  |  |  |  |  |
| 1 | 1,679 (1.8%) | 1,969 (3.4%) | 905 (2.6%) | 2,397 (1.8%) | 2,067 (4.6%) | 89 (1.7%) |
| 2 | 12,219 (13%) | 9,199 (16%) | 4,550 (13%) | 17,284 (13%) | 8,088 (18%) | 596 (11%) |
| 3 | 21,879 (24%) | 14,048 (24%) | 7,783 (22%) | 31,492 (23%) | 11,166 (25%) | 1,052 (20%) |
| 4 | 23,931 (26%) | 14,161 (24%) | 8,574 (24%) | 35,086 (26%) | 10,265 (23%) | 1,315 (25%) |
| 5 | 18,584 (20%) | 10,420 (18%) | 7,331 (21%) | 27,699 (20%) | 7,440 (17%) | 1,196 (23%) |
| >=6 | 14,155 (15%) | 8,159 (14%) | 6,270 (18%) | 21,782 (16%) | 5,770 (13%) | 1,032 (20%) |
| HAS-BLED score | 2.2 (0.6) | 2.2 (0.7) | 2.3 (0.7) | 2.3 (0.6) | 2.2 (0.7) | 2.4 (0.7) |
| Combined comorbidity score | 3.2 (2.4) | 3.2 (2.4) | 3.6 (2.6) | 3.3 (2.5) | 3.1 (2.5) | 3.8 (2.7) |
| Duration of pre-ablation OAC use ^†^ |  |  |  |  |  |  |
| 0 days | 3,257 (3.5%) | 4,060 (7.0%) | 1,664 (4.7%) | 4,903 (3.6%) | 4,078 (9.1%) | 0 (0%) |
| 1-60 days | 8,825 (9.5%) | 8,729 (15%) | 6,601 (19%) | 13,296 (9.8%) | 9,889 (22%) | 970 (18%) |
| 61-180 days | 16,535 (18%) | 13,128 (23%) | 7,917 (22%) | 24,690 (18%) | 11,582 (26%) | 1,308 (25%) |
| 181-364 days | 28,367 (31%) | 19,304 (33%) | 14,529 (41%) | 46,656 (34%) | 12,542 (28%) | 3,002 (57%) |
| 365 days | 35,463 (38%) | 12,735 (22%) | 4,702 (13%) | 46,195 (34%) | 6,705 (15%) | 0 (0%) |
| Frailty category |  |  |  |  |  |  |
| Non-frail | 28,804 (31%) | 18,109 (31%) | 9,264 (26%) | 40,221 (30%) | 14,721 (33%) | 1,235 (23%) |
| Pre-frail | 52,756 (57%) | 32,653 (56%) | 20,117 (57%) | 77,849 (57%) | 24,625 (55%) | 3,052 (58%) |
| Frail | 10,887 (12%) | 7,194 (12%) | 6,032 (17%) | 17,670 (13%) | 5,450 (12%) | 993 (19%) |
| Count of prior major bleeding |  |  |  |  |  |  |
| 0 | 87,571 (95%) | 54,228 (94%) | 32,342 (91%) | 127,938 (94%) | 41,487 (93%) | 4,716 (89%) |
| 1 | 4,269 (4.6%) | 3,243 (5.6%) | 2,602 (7.3%) | 6,824 (5.0%) | 2,822 (6.3%) | 468 (8.9%) |
| >=2 | 607 (0.7%) | 485 (0.8%) | 469 (1.3%) | 978 (0.7%) | 487 (1.1%) | 96 (1.8%) |
| **Comorbidities** |  |  |  |  |  |  |
| Falls | 2,952 (3.2%) | 1,862 (3.2%) | 1,475 (4.2%) | 4,531 (3.3%) | 1,489 (3.3%) | 269 (5.1%) |
| Fractures | 6,059 (6.6%) | 3,978 (6.9%) | 2,880 (8.1%) | 9,392 (6.9%) | 3,056 (6.8%) | 469 (8.9%) |
| Hypertension | 81,966 (89%) | 50,666 (87%) | 31,564 (89%) | 120,949 (89%) | 38,463 (86%) | 4,784 (91%) |
| Obesity | 31,043 (34%) | 18,275 (32%) | 11,374 (32%) | 45,421 (33%) | 13,414 (30%) | 1,857 (35%) |
| Diabetes | 29,535 (32%) | 18,354 (32%) | 12,448 (35%) | 44,322 (33%) | 14,102 (31%) | 1,913 (36%) |
| Peripheral vascular disease | 9,886 (11%) | 6,555 (11%) | 4,810 (14%) | 15,458 (11%) | 5,042 (11%) | 751 (14%) |
| Ischemic stroke | 4,364 (4.7%) | 2,261 (3.9%) | 1,542 (4.4%) | 6,328 (4.7%) | 1,577 (3.5%) | 262 (5.0%) |
| Acute myocardial infarction | 4,646 (5.0%) | 2,975 (5.1%) | 2,216 (6.3%) | 7,125 (5.2%) | 2,370 (5.3%) | 342 (6.5%) |
| Congestive heart failure | 44,559 (48%) | 26,617 (46%) | 18,127 (51%) | 67,309 (50%) | 19,156 (43%) | 2,838 (54%) |
| Valvular heart disease | 45,954 (50%) | 28,230 (49%) | 18,033 (51%) | 68,190 (50%) | 21,281 (48%) | 2,746 (52%) |
| Chronic kidney disease | 15,179 (16%) | 9,767 (17%) | 7,081 (20%) | 23,607 (17%) | 7,233 (16%) | 1,187 (22%) |
| Dementia | 2,461 (2.7%) | 1,526 (2.6%) | 1,318 (3.7%) | 3,912 (2.9%) | 1,190 (2.7%) | 203 (3.8%) |
| COPD | 18,886 (20%) | 12,323 (21%) | 8,770 (25%) | 29,233 (22%) | 9,341 (21%) | 1,405 (27%) |
| COVID | 3,402 (3.7%) | 992 (1.7%) | 863 (2.4%) | 4,362 (3.2%) | 755 (1.7%) | 140 (2.7%) |
| **Medications** |  |  |  |  |  |  |
| ACE inhibitor or ARB | 55,437 (60%) | 33,391 (58%) | 20,862 (59%) | 81,621 (60%) | 24,885 (56%) | 3,184 (60%) |
| Beta blockers | 77,827 (84%) | 47,148 (81%) | 29,095 (82%) | 114,147 (84%) | 35,493 (79%) | 4,430 (84%) |
| Calcium channel blockers | 44,551 (48%) | 27,318 (47%) | 16,953 (48%) | 65,646 (48%) | 20,590 (46%) | 2,586 (49%) |
| Diuretics | 52,794 (57%) | 31,153 (54%) | 20,430 (58%) | 78,625 (58%) | 22,535 (50%) | 3,217 (61%) |
| Lipid lowering drugs | 63,864 (69%) | 38,366 (66%) | 23,841 (67%) | 93,707 (69%) | 28,827 (64%) | 3,537 (67%) |
| Antiarrhythmic agents | 60,083 (65%) | 36,539 (63%) | 21,466 (61%) | 88,107 (65%) | 26,662 (60%) | 3,319 (63%) |
| Rate control agents | 80,537 (87%) | 49,386 (85%) | 30,500 (86%) | 118,234 (87%) | 37,579 (84%) | 4,610 (87%) |
| P2Y12 inhibitor | 9,023 (9.8%) | 6,156 (11%) | 4,231 (12%) | 13,568 (10%) | 5,234 (12%) | 608 (12%) |
| Proton-pump inhibitors | 36,429 (39%) | 21,411 (37%) | 13,179 (37%) | 52,870 (39%) | 16,108 (36%) | 2,041 (39%) |
| Parkinsonism drugs | 3,122 (3.4%) | 2,251 (3.9%) | 1,429 (4.0%) | 4,881 (3.6%) | 1,652 (3.7%) | 269 (5.1%) |
| Opioids | 30,345 (33%) | 21,043 (36%) | 13,471 (38%) | 46,394 (34%) | 16,366 (37%) | 2,099 (40%) |
| **Procedures** | 23,464 (25%) | 13,477 (23%) | 9,236 (26%) | 35,352 (26%) | 9,420 (21%) | 1,405 (27%) |
| Cardiomyopathy | 37,216 (40%) | 21,263 (37%) | 12,592 (36%) | 53,648 (40%) | 15,470 (35%) | 1,953 (37%) |
| Cardioversion | 9,164 (9.9%) | 6,325 (11%) | 3,778 (11%) | 13,513 (10%) | 5,136 (11%) | 618 (12%) |
| Colonoscopy | 74.5 (6.1) | 74.0 (5.9) | 74.8 (6.3) | 74.6 (6.1) | 73.6 (5.8) | 75.1 (6.2) |

Abbreviations: OAC= oral anticoagulants; CHA_2_DS_2_-VASc = Congestive Heart failure (or left ventricular systolic dysfunction), Hypertension (blood pressure consistently >140/90 mm Hg or treated with medication), Age (≥75 y), Diabetes mellitus, Prior Stroke or transient ischemic attack or thromboembolism, Vascular disease (e.g., peripheral artery disease, myocardial infarction, aortic plaque), Age 65-74 y, Sex category (i.e., female sex); HAS-BLED = Hypertension, Abnormal renal/liver function, Stroke, Bleeding history or predisposition, Labile international normalized ratio, Elderly (>65 y), Drugs/alcohol concomitantly; COPD=chronic obstructive pulmonary disease; ACE inhibitor = Angiotensin-Converting Enzyme inhibitor; ARB= Angiotensin II Receptor Blocker; NSAIDs = nonsteroidal anti-inflammatory drugs.

**Table S5**. Post-ablation medications and procedures by pattern groups using anticoagulation discontinuation defined by alternative refill gaps

|  | **30-day gaps** | | | **90-day gaps** | | |
| --- | --- | --- | --- | --- | --- | --- |
|  | **Continued use  N = 92,447** | **Discontinued 3 – 12 months  N = 57,956** | **Discontinued within 3 months  N = 35,413** | **Continued use N = 135,740** | **Discontinued 3 – 12 months  N = 44,796** | **Discontinued within 3 months  N = 5,280** |
| **OAC duration, days** ^*^ | 365 (290, 365) | 276 (176, 337) | 194 (69, 316) | 365 (335, 365) | 229 (161, 306) | 241 (84, 333) |
| **OAC reinitiation** | - | 31,678 (55%) | 24,052 (68%) | - | 12,124 (27%) | 3,709 (70%) |
| **Time to OAC reinitiation, days** ^*, †^ | - | 220 (163, 286) | 79 (50, 113) | - | 246 (187, 306) | 78 (51, 113) |
| **Medications** |  |  |  |  |  |  |
| P2Y12 inhibitors | 5,612 (6.1%) | 6,273 (11%) | 3,736 (11%) | 9,268 (6.8%) | 5,806 (13%) | 547 (10%) |
| Antiarrhythmic agents | 48,194 (52%) | 30,089 (52%) | 16,002 (45%) | 71,180 (52%) | 20,712 (46%) | 2,393 (45%) |
| Rate control agents | 69,081 (75%) | 44,145 (76%) | 26,152 (74%) | 102,807 (76%) | 32,580 (73%) | 3,991 (76%) |
| **Procedures** |  |  |  |  |  |  |
| Cardioversion | 16,461 (18%) | 9,611 (17%) | 5,267 (15%) | 24,574 (18%) | 5,896 (13%) | 869 (16%) |
| Catheter ablation | 23,188 (25%) | 15,243 (26%) | 10,186 (29%) | 36,052 (27%) | 11,002 (25%) | 1,563 (30%) |
| Surgical ablation | 110 (0.1%) | 244 (0.4%) | 140 (0.4%) | 387 (0.3%) | 184 (0.4%) | 23 (0.4%) |
| Left atrial appendage occlusion | 952 (1.0%) | 2,845 (4.9%) | 1,158 (3.3%) | 2,000 (1.5%) | 2,784 (6.2%) | 171 (3.2%) |

Note: ^*^ data is median (IQR); ^†^ Time since last day of supply until a new refill. Abbreviations: OAC= oral anticoagulants.

**Table S6**. Sensitivity analysis of LASSO-selected predictors associated with anticoagulation discontinuation after ablation with discontinuation defined by a 30-day refill gap

|  | **Hazard ratios (95% CI)** | |
| --- | --- | --- |
|  | **Discontinued 3 – 12 months** | **Any Discontinuation** |
| Sex, male | Ref | Ref |
| Sex, female | **0.88 (0.86, 0.90)** | **0.90 (0.89, 0.91)** |
| Region, East | Ref | Ref |
| Region, Midwest | **1.03 (1.00, 1.06)** | **1.03 (1.01, 1.06)** |
| Region, South/Other | **1.13 (1.11, 1.16)** | **1.15 (1.12, 1.17)** |
| Region, West | **1.17 (1.14, 1.20)** | **1.14 (1.12, 1.17)** |
| Year 2013 - 2015 | Ref | Ref |
| Year 2016 - 2019 | **0.90 (0.89, 0.92)** | **0.90 (0.89, 0.92)** |
| Year 2020 - 2022 | **0.89 (0.87, 0.91)** | **0.86 (0.84, 0.87)** |
| CHA_2_DS_2_-VASc score = 1 | Ref | Ref |
| CHA_2_DS_2_-VASc score3 = 2 | **0.77 (0.73, 0.81)** | **0.82 (0.79, 0.86)** |
| CHA_2_DS_2_-VASc score3 >= 3 | **0.69 (0.65, 0.72)** | **0.78 (0.75, 0.81)** |
| Count of prior major bleeding = 0 | Ref | Ref |
| Count of prior major bleeding = 1 | **1.11 (1.07, 1.16)** | **1.14 (1.11, 1.17)** |
| Count of prior major bleeding >= 2 | **1.15 (1.05, 1.25)** | **1.19 (1.12, 1.27)** |
| Duration of pre-ablation OAC use, 0 days | Ref | Ref |
| Duration of pre-ablation OAC use, 1-60 days | **0.88 (0.85, 0.92)** | **1.13 (1.10, 1.17)** |
| Duration of pre-ablation OAC use, 61-180 days | **0.76 (0.73, 0.79)** | **0.94 (0.91, 0.97)** |
| Duration of pre-ablation OAC use, >180 days | **0.66 (0.64, 0.68)** | **0.90 (0.87, 0.93)** |
| Baseline OAC, DOAC | Ref | Ref |
| Baseline OAC, Warfarin | **1.25 (1.22, 1.27)** | **1.23 (1.21, 1.25)** |
| Frailty score | **1.51 (1.28, 1.79)** | **2.31 (2.01, 2.66)** |
| Peripheral vascular disease | Not selected | **1.07 (1.05, 1.09)** |
| Ischemic stroke | Not selected | **0.90 (0.87, 0.93)** |
| Chronic obstructive pulmonary disease | Not selected | **1.04 (1.03, 1.06)** |
| Anemia | **1.05 (1.03, 1.07)** | **1.05 (1.04, 1.07)** |
| Acute renal failure | Not selected | **1.08 (1.06, 1.10)** |
| Cardioversion | **0.90 (0.89, 0.92)** | **0.90 (0.89, 0.91)** |
| Lipid lowering drugs | **0.93 (0.91, 0.95)** | **0.93 (0.92, 0.95)** |
| ACEi or ARB | **0.94 (0.92, 0.95)** | **0.94 (0.93, 0.96)** |
| Beta blockers | **0.92 (0.90, 0.94)** | **0.95 (0.93, 0.97)** |
| Antiarrhythmic agents | Not selected | **0.96 (0.95, 0.98)** |
| Antidepressants | **1.06 (1.04, 1.08)** | **1.03 (1.02, 1.05)** |
| Opioids | **1.06 (1.04, 1.08)** | **1.04 (1.03, 1.06)** |
| Rate control agents | Not selected | **0.95 (0.93, 0.97)** |

**Notes**: Predictors were selected by least absolute shrinkage and selection operator (LASSO) Cox regression. Hazard ratios were estimated using multivariable Cox regression, adjusting for all selected predictors. Statistically significant results are indicated in bold.

**Table S7**. Sensitivity analysis of LASSO-selected predictors associated with anticoagulation discontinuation after ablation with discontinuation defined by a 90-day refill gap

|  | **Hazard ratios (95% CI)** | |
| --- | --- | --- |
|  | **Discontinued 3 – 12 months** | **Any Discontinuation** |
| Age < 75 years | Ref | Not selected |
| Age >=75 years | **0.92 (0.90, 0.94)** | Not selected |
| Sex, male | Ref | Ref |
| Sex, female | **0.80 (0.78, 0.82)** | **0.82 (0.80, 0.83)** |
| Region, East | Ref | Ref |
| Region, Midwest | **1.07 (1.04, 1.11)** | **1.08 (1.04, 1.11)** |
| Region, South/Other | **1.19 (1.16, 1.23)** | **1.19 (1.16, 1.22)** |
| Region, West | **1.27 (1.23, 1.31)** | **1.25 (1.21, 1.29)** |
| Year 2013 - 2015 | Ref | Not selected |
| Year 2016 - 2019 | **0.84 (0.82, 0.86)** | Not selected |
| Year 2020 - 2022 | **0.86 (0.84, 0.88)** | Not selected |
| CHA_2_DS_2_-VASc score = 1 | Ref | Ref |
| CHA_2_DS_2_-VASc score3 = 2 | **0.77 (0.73, 0.81)** | **0.77 (0.74, 0.81)** |
| CHA_2_DS_2_-VASc score3 >= 3 | **0.71 (0.67, 0.74)** | **0.68 (0.65, 0.72)** |
| Count of prior major bleeding = 0 | Ref | Ref |
| Count of prior major bleeding = 1 | **1.24 (1.19, 1.30)** | **1.25 (1.21, 1.30)** |
| Count of prior major bleeding >= 2 | **1.52 (1.38, 1.66)** | **1.52 (1.40, 1.65)** |
| Duration of pre-ablation OAC use, 0 days | Ref | **Ref** |
| Duration of pre-ablation OAC use, 1-60 days | **0.97 (0.94, 1.01)** | **1.06 (1.02, 1.10)** |
| Duration of pre-ablation OAC use, 61-180 days | **0.70 (0.67, 0.72)** | **0.77 (0.75, 0.80)** |
| Duration of pre-ablation OAC use, >180 days | **0.45 (0.43, 0.46)** | **0.55 (0.53, 0.57)** |
| Obesity | **0.96 (0.94, 0.98)** | Not selected |
| Ischemic stroke | **0.86 (0.82, 0.90)** | Not selected |
| Congestive heart failure | **0.95 (0.92, 0.97)** | Not selected |
| Non-ischemic stroke | **0.85 (0.79, 0.90)** | Not selected |
| Anemia | **1.10 (1.08, 1.13)** | **1.11 (1.08, 1.13)** |
| Valvular heart disease | **0.96 (0.94, 0.98)** | Not selected |
| Cardiomyopathy | **0.89 (0.87, 0.92)** | **0.89 (0.87, 0.91)** |
| Cardioversion | **0.88 (0.86, 0.90)** | **0.86 (0.85, 0.88)** |
| Lipid lowering drugs | **0.91 (0.89, 0.93)** | **0.91 (0.89, 0.93)** |
| ACEi or ARB | **0.93 (0.91, 0.94)** | **0.94 (0.92, 0.95)** |
| Beta blockers | **0.92 (0.90, 0.95)** | **0.92 (0.90, 0.94)** |
| Calcium channel blockers | **0.96 (0.95, 0.98)** | Not selected |
| Diuretics | **0.96 (0.94, 0.98)** | **0.96 (0.94, 0.98)** |
| Antiarrhythmic agents | **0.90 (0.88, 0.92)** | **0.92 (0.90, 0.94)** |
| Rate control agents | **0.97 (0.94, 1.00)** | Not selected |
| Opioids | **1.07 (1.05, 1.09)** | **1.08 (1.06, 1.10)** |
| Colonoscopy | **1.05 (1.02, 1.08)** | Not selected |

**Notes**: Predictors were selected by least absolute shrinkage and selection operator (LASSO) Cox regression. Hazard ratios were estimated using multivariable Cox regression, adjusting for all selected predictors. Statistically significant results are indicated in bold.

**Figure S1**. Proportion of patients remaining on anticoagulation after ablation in subgroups by age and sex based on CHA_2_DS_2_-VASc score.

**Figure S2**. Predictors of OAC discontinuation 3 –12 months after ablation in patients with elevated stroke risk (CHA_2_DS_2_-VASc score ≥ 3 for men or ≥ 2 for women). Hazard ratios were estimated using multivariable Cox regression, adjusting for all selected predictors. Abbreviations: OAC=oral anticoagulants; COPD=chronic obstructive pulmonary disease; ACE inhibitor = Angiotensin-Converting Enzyme inhibitor; ARB= Angiotensin II Receptor Blocker.

**Figure S3**. Proportion of patients remaining on anticoagulation after ablation by CHA_2_DS_2_-VASc score. Left panel used discontinuation defined by a 30-day gap and right panel used discontinuation defined by a 60-day gap.

**Supplementary Method**

**Study definitions**

**Exposure Definitions**

| **Exposure** | **Codes/Definitions** |
| --- | --- |
| Catheter ablation | **HCPCS Procedure Code is any of:** 93462, 93650, 93653, 93654, 93655, 93656, 93657  **ICD-9 Procedure Code (any position) is**: 37.90  **ICD-10 Procedure Code (any position) is any of**: 02553ZZ, 02563ZZ, 02573ZZ, 02583ZZ, 02593ZZ, 025F3ZZ, 025G3ZZ, 025H3ZZ, 025J3ZZ, 025K3ZZ, 025L3ZZ, 025M3ZZ, 02B53ZZ, 02B63ZZ, 02B73ZZ, 02B83ZZ, 02B93ZZ, 02BF3ZZ, 02BG3ZZ, 02BH3ZZ, 02BJ3ZZ, 02BK3ZZ, 02BL3ZZ, 02BM3ZZ, 02T83ZZ |
| **Oral anticoagulants** | **Codes/Definitions** |
| Warfarin | **NDC Generic Name:** WARFARIN SODIUM |
| Apixaban | **NDC Generic Name:** APIXABAN |
| Rivaroxaban | **NDC Generic Name:** RIVAROXABAN |
| Dabigatran | **NDC Generic Name:** DABIGATRAN ETEXILATE MESYLATE |
| Edoxaban | **NDC Generic Name:** EDOXABAN TOSYLATE |

**Medication & Procedure Definitions**

| Surgical ablation | **HCPCS Procedure Code is any of** 33250, 33251, 33254, 33255, 33256, 33257, 33258, 33259, 33261  **ICD-9 Procedure Code (any position) is**: 37.33  **ICD-10 Procedure Code (any position) is any of**: 02550ZZ, 02560ZZ, 02570ZZ, 02580ZZ, 02590ZZ, 025F0ZZ, 025G0ZZ, 025H0ZZ, 025J0ZZ, 025K0ZZ, 025L0ZZ, 025M0ZZ, 02B50ZZ, 02B60ZZ, 02B70ZZ, 02B80ZZ, 02B90ZZ, 02BF0ZZ, 02BG0ZZ, 02BH0ZZ, 02BJ0ZZ, 02BK0ZZ, 02BL0ZZ, 02BM0ZZ, 02T80ZZ |
| --- | --- |
| Cardioversion | **HCPCS Procedure Code is any of** 92960, 92961  **ICD-9 Procedure Code (any position) is**: 99.61  **ICD-10 Procedure Code (any position) is any of**: 5A2204Z |
| Antiarrhythmic Drug | **NDC Generic Name:** AMIODARONE HCL, DOFETILIDE, DRONEDARONE HCL, FLECAINIDE ACETATE, PROPAFENONE HCL, SOTALOL HCL |
| Rate Control Beta blockers Agents | **NDC Generic Name:** METOPROLOL TARTRATE, METOPROLOL SUCCINATE, ATENOLOL, BISOPROLOL FUMARATE, CARVEDILOL  CARVEDILOL PHOSPHATE, ESMOLOL HCL, ESMOLOL HCL IN SODIUM CHLORIDE, ISO-OSMOTIC, ESMOLOL HCL IN STERILE WATER  NADOLOL, PROPRANOLOL HCL |
| Rate Control Calcium Channel Blocker Agents | **NDC Generic Name:** DILTIAZEM HCL, DILTIAZEM HCL IN SODIUM CHLORIDE, ISO-OSMOTIC, DILTIAZEM HCL/DEXTROSE 5 % IN WATER, DILTIAZEM MALATE, VERAPAMIL HCL |
| Digoxin | **NDC Generic Name:** DIGOXIN, DIGOXIN IMMUNE FAB |
| P2Y12 inhibitor | **NDC Generic Name:** CLOPIDOGREL BISULFATE, PRASUGREL HCL, TICAGRELOR |

**Event definitions**

| **Outcome** | **Codes/Definitions** |
| --- | --- |
| Major Bleeding event | **Primary position, inpatient ICD-9 Diagnosis Code is any of**: 336.1, 363.61, 363.72, 376.32, 430, 432, 432.9, 568.81, 719.1, 719.10, 719.15, 719.18, 852, 852.0, 852.00, 852.04, 852.05, 852.10, 852.11, 852.12, 852.16, 852.20, 852.21, 852.22, 852.23, 852.25, 852.29, 852.3, 852.31, 852.32, 852.35, 852.36, 852.40, 852.43, 852.5, 852.51, 852.52, 852.53, 852.54, 852.59, 853.0, 853.00, 853.04, 853.05, 853.09, 853.10, 853.11, 853.13, 853.19, 866.02, 866.11, 866.12, 363.62, 377.42, 379.23, 423.0, 431, 432.0, 432.1, 719.11, 719.12, 719.13, 719.14, 719.16, 719.17, 719.19, 729.92, 852.01, 852.02, 852.03, 852.06, 852.09, 852.1, 852.13, 852.14, 852.15, 852.19, 852.2, 852.24, 852.26, 852.30, 852.33, 852.34, 852.39, 852.4, 852.41, 852.42, 852.44, 852.45, 852.46, 852.49, 852.50, 852.55, 852.56, 853, 853.01, 853.02, 853.03, 853.06, 853.1, 853.12, 853.14, 853.15, 853.16, 866.01  **Primary position, inpatient ICD-10 Diagnosis Code is any of**: G95.19, H05.23, H05.231, H05.232, H05.239, H31.303, H31.311, H31.312, H31.319, H31.412, H31.419, H43.11, H43.12, H47.029, I31.2, I60.0, I60.01, I60.10, I60.11, I60.3, I60.31, I60.5, I60.51, I60.52, I60.6, I60.8, I61, I61.4, I61.8, I62.1, K66.1, M25.011, M25.012, M25.02, M25.022, M25.03, M25.032, M25.04, M25.041, M25.042, M25.049, M25.05, M25.059, M25.06, M25.061, M25.062, M25.069, M25.071, M25.074, M25.076, M25.08, S06.4X0D, S06.4X0S, S06.4X1, S06.4X1S, S06.4X2, S06.4X2D, S06.4X3, S06.4X3A, S06.4X4, S06.4X4A, S06.4X5S, S06.4X6, S06.4X6A, S06.4X6D, S06.4X6S, S06.4X7, S06.4X7A, S06.4X8A, S06.5X, S06.5X0, S06.5X0S, S06.5X1, S06.5X1A, S06.5X1S, S06.5X2, S06.5X2D, S06.5X2S, S06.5X3, S06.5X3A, S06.5X3D, S06.5X3S, S06.5X4, S06.5X4A, S06.5X4D, S06.5X5D, S06.5X6A, S06.5X6S, S06.5X8, S06.5X8A, S06.5X9A, S06.6, S06.6X0, S06.6X0D, S06.6X1, S06.6X1A, S06.6X1S, S06.6X2, S06.6X2D, S06.6X2S, S06.6X3, S06.6X3A, S06.6X3D, S06.6X4S, S06.6X5A, S06.6X5S, S06.6X6D, S06.6X7A, S06.6X8, S06.6X8A, S06.6X9S, S26.00, S26.00XS, S26.01, S26.01XS, S26.020D, S26.021, S26.021D, S26.021S, S26.022, S26.022A, S26.022S, S26.09XA, S26.09XD, S37.01, S37.011S, S37.012D, S37.021, S37.021A, S37.021D, S37.022, S37.029D, S37.029S, S37.03, S37.031, S37.031A, S37.031S, S37.032S, S37.039D, S37.041S, S37.042, S37.042A, S37.049A, S37.051A, S37.051D, S37.051S, S37.052D, S37.052S, S37.059, S37.059D, S37.06, S37.061, S37.061D, S37.062, S37.062A, S37.062D, S37.062S, S37.069, S37.069A, S37.069S, H05.233, H31.30, H31.301, H31.302, H31.309, H31.31, H31.313, H31.41, H31.411, H31.413, H43.1, H43.10, H43.13, H47.02, H47.021, H47.022, H47.023, I23.0, I60, I60.00, I60.02, I60.1, I60.12, I60.2, I60.30, I60.32, I60.4, I60.50, I60.7, I60.9, I61.0, I61.1, I61.2, I61.3, I61.5, I61.6, I61.9, I62, I62.0, I62.00, I62.01, I62.02, I62.03, I62.9, M25.0, M25.00, M25.01, M25.019, M25.021, M25.029, M25.031, M25.039, M25.051, M25.052, M25.07, M25.072, M25.073, M25.075, S06.4, S06.4X, S06.4X0, S06.4X0A, S06.4X1A, S06.4X1D, S06.4X2A, S06.4X2S, S06.4X3D, S06.4X3S, S06.4X4D, S06.4X4S, S06.4X5, S06.4X5A, S06.4X5D, S06.4X8, S06.4X9, S06.4X9A, S06.4X9D, S06.4X9S, S06.5, S06.5X0A, S06.5X0D, S06.5X1D, S06.5X2A, S06.5X4S, S06.5X5, S06.5X5A, S06.5X5S, S06.5X6, S06.5X6D, S06.5X7, S06.5X7A, S06.5X9, S06.5X9D, S06.5X9S, S06.6X, S06.6X0A, S06.6X0S, S06.6X1D, S06.6X2A, S06.6X3S, S06.6X4, S06.6X4A, S06.6X4D, S06.6X5, S06.6X5D, S06.6X6, S06.6X6A, S06.6X6S, S06.6X7, S06.6X9, S06.6X9A, S06.6X9D, S26.0, S26.00XA, S26.00XD, S26.01XA, S26.01XD, S26.02, S26.020, S26.020A, S26.020S, S26.021A, S26.022D, S26.09, S26.09XS, S37.011, S37.011A, S37.011D, S37.012, S37.012A, S37.012S, S37.019, S37.019A, S37.019D, S37.019S, S37.02, S37.021S, S37.022A, S37.022D, S37.022S, S37.029, S37.029A,  S37.031D, S37.032, S37.032A, S37.032D, S37.039, S37.039A, S37.039S, S37.04, S37.041, S37.041A, S37.041D, S37.042D, S37.042S, S37.049, S37.049D, S37.049S, S37.05, S37.051, S37.052, S37.052A, S37.059A, S37.059S, S37.061A, S37.061S, S37.069D, M79.81  **ICD-9 Procedure Code (any position) is any of: 99.04, 99.05, 99.03, 99.06 with Primary ICD-9 Diagnosis Code is any of:** 285.1, 455.2, 455.5, 455.6, 455.7, 455.8, 456.0, 530.1, 530.11, 530.13, 530.19, 530.7, 531.3, 531.40, 531.5, 531.60, 531.61, 532.20, 532.3, 532.4, 532.41, 532.5, 532.6, 532.61, 532.9, 533.0, 533.00, 533.01, 533.20, 533.3, 533.40, 533.6, 533.60, 533.61, 533.7, 534.01, 534.2, 534.3, 534.4, 534.40, 534.41, 534.5, 534.6, 534.9, 535.00, 535.11, 535.20, 535.30, 535.41, 535.50, 535.61, 562.00, 562.02, 562.03, 562.10, 562.11, 562.12, 562.13, 569.3, 569.85, 578.1, 599.70, 599.71, 623.6, 623.8, 626.6, 626.8, 784.7, 784.8, 786.3, 786.30, 786.39, 280.0, 455.1, 455.4, 455.9, 456.20, 459.0, 530.10, 530.12, 530.82, 531.0, 531.00, 531.01, 531.1, 531.2, 531.20, 531.21, 531.4, 531.41, 531.6, 531.7, 531.9, 532.0, 532.00, 532.01, 532.1, 532.2, 532.21, 532.40, 532.60, 532.7, 533.1, 533.2, 533.21, 533.4, 533.41, 533.5, 533.9, 534.0, 534.00, 534.1, 534.20, 534.21, 534.60, 534.61, 534.7, 535.01, 535.10, 535.21, 535.31, 535.40, 535.51, 535.60, 537.83, 562.01, 578.0, 578.9, 593.81, 786.31  **HCPCS Procedure Code (any position) is any of:** P9010, P9016, P9017, P9019, P9022, P9032, P9036, P9038, P9039, P9051, P9052, P9054, P9055, P9056, P9058, P9059, P9060, P9011, P9020, P9021, P9023, P9031, P9033, P9034, P9035, P9037, P9040, P9044, P9053, P9057, P9070, P9071 **with Primary ICD-9 Diagnosis Code is any of:** 285.1, 455.2, 455.5, 455.6, 455.7, 455.8, 456.0, 530.1, 530.11, 530.13, 530.19, 530.7, 531.3, 531.40, 531.5, 531.60, 531.61, 532.20, 532.3, 532.4, 532.41, 532.5, 532.6, 532.61, 532.9, 533.0, 533.00, 533.01, 533.20, 533.3, 533.40, 533.6, 533.60, 533.61, 533.7, 534.01, 534.2, 534.3, 534.4, 534.40, 534.41, 534.5, 534.6, 534.9, 535.00, 535.11, 535.20, 535.30, 535.41, 535.50, 535.61, 562.00, 562.02, 562.03, 562.10, 562.11, 562.12, 562.13, 569.3, 569.85, 578.1, 599.70, 599.71, 623.6, 623.8, 626.6, 626.8, 784.7, 784.8, 786.3, 786.30, 786.39, 280.0, 455.1, 455.4, 455.9, 456.20, 459.0, 530.10, 530.12, 530.82, 531.0, 531.00, 531.01, 531.1, 531.2, 531.20, 531.21, 531.4, 531.41, 531.6, 531.7, 531.9, 532.0, 532.00, 532.01, 532.1, 532.2, 532.21, 532.40, 532.60, 532.7, 533.1, 533.2, 533.21, 533.4, 533.41, 533.5, 533.9, 534.0, 534.00, 534.1, 534.20, 534.21, 534.60, 534.61, 534.7, 535.01, 535.10, 535.21, 535.31, 535.40, 535.51, 535.60, 537.83, 562.01, 578.0, 578.9, 593.81, 786.31  **Revenue Center Code is any of:** 0387, 0391, 0381, 0382, 0383, 0384 **with Primary ICD-9 Diagnosis Code is any of:** 285.1, 455.2, 455.5, 455.6, 455.7, 455.8, 456.0, 530.1, 530.11, 530.13, 530.19, 530.7, 531.3, 531.40, 531.5, 531.60, 531.61, 532.20, 532.3, 532.4, 532.41, 532.5, 532.6, 532.61, 532.9, 533.0, 533.00, 533.01, 533.20, 533.3, 533.40, 533.6, 533.60, 533.61, 533.7, 534.01, 534.2, 534.3, 534.4, 534.40, 534.41, 534.5, 534.6, 534.9, 535.00, 535.11, 535.20, 535.30, 535.41, 535.50, 535.61, 562.00, 562.02, 562.03, 562.10, 562.11, 562.12, 562.13, 569.3, 569.85, 578.1, 599.70, 599.71, 623.6, 623.8, 626.6, 626.8, 784.7, 784.8, 786.3, 786.30, 786.39, 280.0, 455.1, 455.4, 455.9, 456.20, 459.0, 530.10, 530.12, 530.82, 531.0, 531.00, 531.01, 531.1, 531.2, 531.20, 531.21, 531.4, 531.41, 531.6, 531.7, 531.9, 532.0, 532.00, 532.01, 532.1, 532.2, 532.21, 532.40, 532.60, 532.7, 533.1, 533.2, 533.21, 533.4, 533.41, 533.5, 533.9, 534.0, 534.00, 534.1, 534.20, 534.21, 534.60, 534.61, 534.7, 535.01, 535.10, 535.21, 535.31, 535.40, 535.51, 535.60, 537.83, 562.01, 578.0, 578.9, 593.81, 786.31  **ICD-10 Procedure Code (any position) is any of:** 30230P1, 30230T1, 30233H1, 30233M1, 30233R1, 30233W1, 30240K1, 30240W1, 30243H1, 30243M1, 30243N1, 30243R1, 30243T1, 30243V1, 30230H1, 30230K1, 30230L1, 30230M1, 30230N1, 30230R1, 30230V1, 30230W1, 30233K1, 30233L1, 30233N1, 30233P1, 30233T1, 30233V1, 30240H1, 30240L1, 30240M1, 30240N1, 30240P1, 30240R1, 30240T1, 30240V1, 30243K1, 30243L1, 30243P1, 30243W1 with Primary ICD-10 Diagnosis Code is any of: D50.0, D62, I85.11, K20, K20.8, K21.0, K22.11, K22.6, K25.0, K25.4, K25.6, K26.1, K26.4, K26.5, K26.6, K27.2, K27.5, K27.6, K28.0, K28.4, K28.5, K29.01, K29.21, K29.41, K31.811, K55.21, K57.01, K57.21, K57.51, K57.53, K57.93, K64.0, K64.5, K64.8, K64.9, K92.0, N89.7, N92.1, N93.9, R04.0, R04.1, R04.2, R31.0, R31.9, R58, I85.01, K20.0, K20.9, K25.1, K25.2, K25.5, K26.0, K26.2, K27.0, K27.1, K27.4, K28.1, K28.2, K28.6, K29.31, K29.51, K29.61, K29.71, K29.81, K29.91, K57.11, K57.31, K57.41, K57.81, K57.91, K62.5, K64.1, K64.2, K64.3, K64.4, K92.1, K92.2, N89.8, N93.8  **HCPCS Procedure Code (any position) is any of:** P9010, P9016, P9017, P9019, P9022, P9032, P9036, P9038, P9039, P9051, P9052, P9054, P9055, P9056, P9058, P9059, P9060, P9011, P9020, P9021, P9023, P9031, P9033, P9034, P9035, P9037, P9040, P9044, P9053, P9057, P9070, P9071 **with Primary ICD-10 Diagnosis Code is any of:** D50.0, D62, I85.11, K20, K20.8, K21.0, K22.11, K22.6, K25.0, K25.4, K25.6, K26.1, K26.4, K26.5, K26.6, K27.2, K27.5, K27.6, K28.0, K28.4, K28.5, K29.01, K29.21, K29.41, K31.811, K55.21, K57.01, K57.21, K57.51, K57.53, K57.93, K64.0, K64.5, K64.8, K64.9, K92.0, N89.7, N92.1, N93.9, R04.0, R04.1, R04.2, R31.0, R31.9, R58, I85.01, K20.0, K20.9, K25.1, K25.2, K25.5, K26.0, K26.2, K27.0, K27.1, K27.4, K28.1, K28.2, K28.6, K29.31, K29.51, K29.61, K29.71, K29.81, K29.91, K57.11, K57.31, K57.41, K57.81, K57.91, K62.5, K64.1, K64.2, K64.3, K64.4, K92.1, K92.2, N89.8, N93.8  **Revenue Center Code is any of:** 0387, 0391, 0381, 0382, 0383, 0384 **with Primary ICD-10 Diagnosis Code is any of:** D50.0, D62, I85.11, K20, K20.8, K21.0, K22.11, K22.6, K25.0, K25.4, K25.6, K26.1, K26.4, K26.5, K26.6, K27.2, K27.5, K27.6, K28.0, K28.4, K28.5, K29.01, K29.21, K29.41, K31.811, K55.21, K57.01, K57.21, K57.51, K57.53, K57.93, K64.0, K64.5, K64.8, K64.9, K92.0, N89.7, N92.1, N93.9, R04.0, R04.1, R04.2, R31.0, R31.9, R58, I85.01, K20.0, K20.9, K25.1, K25.2, K25.5, K26.0, K26.2, K27.0, K27.1, K27.4, K28.1, K28.2, K28.6, K29.31, K29.51, K29.61, K29.71, K29.81, K29.91, K57.11, K57.31, K57.41, K57.81, K57.91, K62.5, K64.1, K64.2, K64.3, K64.4, K92.1, K92.2, N89.8, N93.8 |
| Ischemic Stroke | **Primary position, inpatient ICD-9 Diagnosis Code is any of:** 433.01, 433.11, 433.21, 433.31, 434.01, 434.11, 362.31, 362.32, 362.33, 362.34, 433.81, 433.91, 436  **Primary ICD-10 Diagnosis Code is any of:** H34.0, H34.00, H34.01, H34.02, H34.03, H34.1, H34.10, H34.11, H34.13, H34.2, H34.21, H34.213, H34.23, H34.231, H34.239, I63.011, I63.012, I63.031, I63.112, I63.12, I63.132, I63.139, I63.19, I63.2, I63.21, I63.212, I63.213, I63.219, I63.22, I63.231, I63.232, I63.233, I63.239, I63.29, I63.30, I63.31, I63.311, I63.32, I63.322, I63.329, I63.33, I63.339, I63.34, I63.341, I63.342, I63.349, I63.39, I63.40, I63.411, I63.419, I63.422, I63.429, I63.431, I63.433, I63.49, I63.50, I63.51, I63.52, I63.523, I63.529, I63.531, I63.533, I63.541, I63.542, I63.543, I63.8, I63.9, H34.12, H34.211, H34.212, H34.219, H34.232, H34.233, I63, I63.0, I63.00, I63.01, I63.013, I63.019, I63.02, I63.03, I63.032, I63.033, I63.039, I63.09, I63.1, I63.10, I63.11, I63.111, I63.113, I63.119, I63.13, I63.131, I63.133, I63.20, I63.211, I63.23, I63.3, I63.312, I63.313, I63.319, I63.321, I63.323, I63.331, I63.332, I63.333, I63.343, I63.4, I63.41, I63.412, I63.413, I63.42, I63.421, I63.423, I63.43, I63.432, I63.439, I63.44, I63.441, I63.442, I63.443, I63.449, I63.5, I63.511, I63.512, I63.513, I63.519, I63.521, I63.522, I63.53, I63.532, I63.539, I63.54, I63.549, I63.59, I63.6 |
| All-Cause Mortality | **Death recorded by Master Beneficiary Summary File or Inpatient Discharge Status Code is any of:** 20, 40, 41, 42 |

**Inclusion/Exclusion Definitions**

| **Inclusion/Exclusion** | **Codes/Definitions** |
| --- | --- |
| Atrial fibrillation | **Inpatient ICD-9 Diagnosis Code (any position) is any of:** 427.3, 427.31  **Inpatient ICD-10 Diagnosis Code (any position) is any of**: I48.0, I48.1, I48.2, I48.91, I48.11, I48.19, I48.20, I48.21, I48, I48.9  **Outpatient ICD-9 Diagnosis Code (any position) is any of:** 427.3, 427.31, **with another diagnosis 7 days – 365 days prior**  **Outpatient ICD-10 Diagnosis Code (any position) is any of:** I48.0, I48.1, I48.2, I48.91, I48.11, I48.19, I48.20, I48.21, I48, I48.9, **with another diagnosis 7 days – 365 days prior** |
| Pulmonary Embolism | **ICD-9 Diagnosis Code (any position) is any of:** 415.11, 415.12, 415.19, 415.1, 415.10, 415.199, 415.15, 415.16, 415.17, 415.13  **ICD-10 Diagnosis Code (any position) is any of:** I26.01, I26.09, I26.92, I26.0, I26.99, I26.02, I26.9, I26.90 |
| Deep Vein Thrombosis | **ICD-9 Diagnosis Code (any position) is any of:** 451.0, 451.1, 451.11, 451.19, 451.2, 451.8, 451.81, 451.82, 451.83, 451.84, 451.89, 451.9, 453.0, 453.1, 453.2, 453.3, 453.4, 453.40, 453.41, 453.42, 453.5, 453.50, 453.51, 453.52, 453.6, 453.7, 453.71, 453.72, 453.73, 453.74, 453.75, 453.76, 453.77, 453.79, 453.8, 453.81, 453.82, 453.83, 453.84, 453.85, 453.86, 453.87, 453.89, 453.9  **ICD-10 Diagnosis Code (any position) is any of:** I80.222, I80.229, I80.232, I80.233, I80.29, I80.293, I82.21, I82.220, I82.29, I82.290, I82.402, I82.43, I82.432, I82.443, I82.491, I82.4Y3, I82.602, I82.611, I82.90, I82.A19, I82.B1, I80.21, I80.9, I82.401, I82.409, I82.411, I82.419, I82.42, I82.421, I82.422, I82.429, I82.439, I82.44, I82.441, I82.492, I82.4Z, I82.4Z3, I82.612, I82.619, I82.622, I82.B11, I82.C11, I82.C13, I82.C19, I80.201,I80.203, I80.211, I80.212, I80.213, I80.22, I80.223, I80.23, I80.231, I82.22, I82.4, I82.403, I82.412, I82.442, I82.493, I82.4Z2, I82.60, I82.601, I82.613, I82.623, I82.890, I82.A11, I82.A12, I82.A13, I80.2, I80.20, I80.219, I80.221, I80.291, I82.1, I82.2, I82.40, I82.413, I82.431, I82.433, I82.4Y1, I82.4Y9, I82.4Z9, I82.61, I82.62, I82.B13, I82.B19, I82.C12, I80.202, I80.209, I80.239, I80.292, I80.299, I80.3, I82.0, I82.210, I82.41, I82.423, I82.449, I82.49, I82.499, I82.4Y, I82.4Y2, I82.4Z1, I82.6, I82.603, I82.609, I82.621, I82.629, I82.A1, I82.B12, I82.C1  **ICD-10 Diagnosis Code (any position) is any of:** T81.718A, T80.0XXA, T81.72XA, T82.817A, T82.818A **with ICD-10 Diagnosis Code (any position) is any of**: I26.99, I26.90 |
| Mitral Stenosis | **ICD-9 Diagnosis Code (any position) is any of:** 394.0  **ICD-10 Diagnosis Code (any position) is any of:** I05.0 |
| Mitral stenosis with insufficiency | **ICD-9 Diagnosis Code (any position) is any of:** 394.2  **ICD-10 Diagnosis Code (any position) is any of**: I05.2 |
| Mechanical valve | **ICD-9 Diagnosis Code (any position) is any of:** V43.3  **ICD-10 Diagnosis Code (any position) is any of:** Z95.2  **ICD-9 Procedure Code (any position) is any of:** 35.22, 35.24  **ICD-10 Procedure Code (any position) is any of:** 02RF0JZ, 02RF4JZ, 02RG0JZ, 02RG3JZ, 02RG4JZ |
| Hospice Stay | Hospice Stay Hospice claim or Inpatient stay with Patient Discharge Status = 50 or 51 |
| End stage live disease | **ICD-9 Diagnosis Code (any position) is any of:** 456.0, 456.20, 789.5, 789.51, 789.57, 789.58, 789.59, 572.2, 572.4, 572.3, 567.23, 571.20, 572.3, 571.5, 571.50, 571, 571.2, 571.6  **ICD-10 Diagnosis Code (any position) is any of:** I85.11, I85.01, R18, R18.0, R18.8,  G92, G93.4, G93.40, G93.41, G93.49, K76.7, K76.6, K65.2, K74.6, K74.60, K74.69, K76.6, K70.3, K70.30, K70.31, K71.7, K72.1, K72.10, K72.11, K74.3, K74.4, K74.5 |
| End stage renal disease or dialysis | **ICD-9 Diagnosis Code (any position) is any of:** V45.1, V45.10, V45.11, V45.111, V45.12, V45.13, V45.15, V56.0, V56.00, V56.8, V56.81, V42.0, 996.81, 585.5, 585.50, 585.53, 585.56, 585.6, V56.1, V56.2, V56.31, V56.32  **HCPCS Procedure Code (any position) is any of:** 50360, 50365, 50380, 90935, 90937, 90940, 90945, 90947, 90989, 90993, 99512, 99559  **ICD-9 Procedure Code (any position) is any of:** 39.95, 54.98  **Line Place Of Service Code is not any of:** 31, 32, 23, 42, **with HCPCS Procedure Code is any of:** 90935, 90937, 90945, 90947 |
